# Global psychological assessment with the evaluation of life and sleep quality and sexual and cognitive function in a large number of patients with acromegaly: a cross-sectional study

**DOI:** 10.1530/EJE-22-0263

**Published:** 2022-09-27

**Authors:** Rosario Pivonello, Renata Simona Auriemma, Alessandra Delli Veneri, Francesca Dassie, Riccardina Lorusso, Marta Ragonese, Marco Liotta, Elisa Sala, Barbara Zarino, Elisa Lai, Claudio Urbani, Fausto Bogazzi, Giovanna Mantovani, Salvatore Cannavò, Pietro Maffei, Paolo Chiodini, Annamaria Colao

**Affiliations:** 1 Dipartimento di Medicina Clinica e Chirurgia, Sezione di Endocrinologia, Università Federico II di Napoli, Naples, Italy; 2 UNESCO Chair for Health Education and Sustainable Development, ‘Federico II’ University, Naples, Italy; 3 Department of Medicine, Clinica Medica 3^, University of Padua, Padua, Italy; 4 Endocrine Unit, University Hospital ‘G. Martino’, Messina, Italy; 5 Endocrinology Unit; 6 Neurosurgery Unit, Fondazione IRCCS Ca’ Granda Ospedale Maggiore Policlinico, Milan, Italy; 7 Psychology Unit, Department of Surgical, Medical, Molecular, and Critical Area Pathology, University of Pisa, Pisa, Italy; 8 Endocrinology II Unit, Department of Medicine, Azienda Ospedaliero Universitaria Pisana, Pisa, Italy; 9 Endocrinology Unit, Department of Clinical and Experimental Medicine, University of Pisa, Pisa, Italy; 10 Department of Clinical Sciences and Community Health, University of Milan, Milan, Italy; 11 Medical Statistic Unit, University of Campania ‘Luigi Vanvitelli’, Naples, Italy

## Abstract

**Objective:**

Acromegaly is associated with somatic disfigurements which impair self-perception of well-being and quality of life. Nowadays, limited data are available on the interplay between hormonal excess and psychological discomfort. The study aimed at investigating the psychological profile, sleep quality, sexual function, cognitive functions, and quality of life in patients with acromegaly.

**Methods:**

In 223 acromegaly patients from 5 referral centres, global psychological profile, sleep quality, sexual function, cognitive function, and quality of life were investigated.

**Results:**

Depression was found in ~30% of patients, and anxiety in two-thirds, together with severe discomfort in body image mainly in women. Obstructive sleep apnoea syndrome risk and sleep disorders were found in >50% of patients and daily sleepiness in ~20%. Sexual dysfunction was reported in most of the patients, with the most severe impairment in women. Cognitive functions were compromised in ~10% of cases. Disease duration and patient’s age and gender were the main determinants of these psychopathological conditions. Depression (*P* = 0.047), somatic-affective mood lowering (*P* = 0.021), state (*P* < 0.001) and trait (*P* = 0.013) anxiety, and body image distortion in body uneasiness test A (*P* < 0.001) and B (*P* = 0.006) were significantly worsened in patients <45 years and slightly worsened in those with disease duration less than 2 years. Male (*P* < 0.001) and female (*P* < 0.001) sexual function scores were significantly worsened in patients aged >64 years and slightly worsened in those with disease duration for more than 10 years, particularly in presence of cardiometabolic and respiratory complications. Cognitive symptoms were slightly worsened in older patients and in those with long disease duration.

**Conclusions:**

Acromegaly is associated with a relevant impairment of psychological profile persisting despite remission and long-term medical treatment.

## Introduction

Acromegaly is a slowly progressive disease resulting from the increased release of growth hormone (GH) and, consequently, insulin-like growth factor I (IGF1), which in most cases is induced by a GH-secreting pituitary tumour (1, 2). Prolonged exposure to hormone excess induces progressive somatic disfigurement and a wide range of systemic manifestations (1, 2, 3, 4, 5). The most common complications associated with acromegaly include cardiovascular, respiratory, and metabolic comorbidities which are among the main clinical conditions responsible for the increased mortality associated with the disease (3, 4, 5). However, similarly to several organic dimensions, especially in the case of chronic and debilitating diseases, the physical burden of acromegaly results in a remarkable psychological impact because of the strict interconnection between body and mind (3, 4). On the other hand, a close relationship between neuroendocrine system and mental disorders has been demonstrated in patients with depression (6). Psychological factors, such as depression and anxiety, seem to be crucial in the clinical course of the chronic and debilitating acromegalic disease. Indeed, acromegalic patients gradually and inexorably experience body changes and loss of control on their body, which may be perceived as a relevant transformation during the disease evolution, till a complete body disfigurement, increasing the risk of psychological morbidity (3, 4). A recent study highlighted that the marked reduction of quality of life (QoL) in acromegaly is driven dominantly by psychopathology rather than biochemical control of the disease, recommending systematic screening for psychopathology and specific psychological therapy in acromegaly to improve patients’ QoL (7). Noteworthy, patients treated for endocrine diseases can frequently experience psychological distress, even after adequate treatment (8, 9). More specifically, acromegaly patients with long-term cure have a high prevalence of psychopathology, including major depression, anxiety, and affective disorders, together with irritability, impatience, and loss of drive, and a greater degree of maladaptive personality traits as compared to matched controls and to patients with non-functioning pituitary tumours (10). Conflicting outcomes have emerged on cognitive functions in patients with acromegaly. Some studies reported normal cognition in patients with long-term cured acromegaly, while some different studies showed attention and memory deficits, although a key role of emotions on cognitive management has been confirmed (11, 12). In particular, an impairment of attention, memory, and executive functions was found in up to 33, 24, and 17%, respectively, of acromegaly patients in association with a decrease in the neural activity in specific brain areas (13, 14). Sleep disorders have been extensively explored in acromegaly considering the influence of obstructive sleep apnoea syndrome (OSAS), a frequent severe complication of the disease, together with the impairment of cognitive function, on general sleep quality (15, 16, 17), testifying that the impairment of the sleep quality was associated with the cognitive dysfunction. Sexual function remains substantially underexplored and underestimated in acromegaly (18, 19, 20, 21, 22, 23, 24), while the role of GH–IGF1 axis has been described on sexuality. GH is an important regulator of the hypothalamus–pituitary–gonadal axis and seems to participate in the sexual response in men and women. A general decrease of desire and arousal in both sexes, together with impairment of erectile function in men, have been described in patients with acromegaly, although it is not clear whether they are dependent directly on the hormone excess or are a consequence of the hypogonadism and/or the different clinical complications and/or the physical disfigurement with consequent psychological imbalance, which are associated with the disease (18, 19, 20, 21, 22, 23, 24). Overall, the well-known body impairment, typical of the acromegalic disease, reportedly exerts a remarkable negative impact on everyday life and global QoL (25, 26). In general, the impact of the prolonged and sustained GH and IGF1 excess, and particularly, the impact of the long-term control of the GH–IGF1 excess, on the psychological burden of acromegaly is unclear. The literature on the general psychological assessment and specific psychopathological conditions in acromegaly is still limited, and the interplay between GH and IGF1 excess and psychological discomfort is yet to be clarified. The current study aims at assessing the psychological profile of surgically or medically treated acromegaly patients, including the sleep quality, the cognitive and sexual functions, and the QoL, and at investigating whether these psychopathological dimensions are associated with age and gender and/or affected by specific illness traits, such as GH and IGF1 values, disease duration, and the co-occurrence of specific comorbidities associated with acromegalic disease.

## Patients and methods

### Inclusion and exclusion criteria

From 2017 to 2019, the study was conducted in five Italian referral centres. The study included adult patients with acromegalic disease after surgical or under medical treatment. Exclusion criteria were: (i) drugs and alcohol abuse, (ii) neurological diseases, and (iii) psychiatric diseases. The psychiatric diseases were considered exclusion criteria to avoid the risk of overlap between the neuropsychiatric diagnosis of depression or anxiety diseases (or their comorbidity with different psychiatric and neurological conditions) with the depression or anxiety syndromes associated with acromegaly. Moreover, patients with the diagnosis of acromegaly at the time of the recruitment (naïve) have been excluded from the analysis performed in the current study.

Overall, 223 patients (94 men, 129 women) with acromegaly, including 165 under medical treatment and 58 in surgical remission, met the eligibility criteria for the current study. Demographic characteristics are presented in Table 1. The whole cohort age range was 18–85 years, with a median age of 56 years (interquartile range (IQR) 48; 64.5). According to age tertiles, patients were categorized as <45 years, 45–64 years, and >64 years. The presumed duration of acromegaly was assessed by comparing photographs taken over a period of 10–30 years and by interviewing the patients as to the date of onset of acral enlargement and facial disfigurement. The median disease duration was 5.0 (IQR 2.0; 10.0) years. Disease duration was categorized as <2 years, 2–5 years, 5–10 years, and >10 years. The diagnosis of acromegaly was based on the following criteria: (i) lack of GH suppression below 1.0 μg/L after a 75 g oral glucose load; (ii) IGF1 levels above the upper limit of normal age range; and (iii) pituitary tumour confirmed at pituitary MRI. At the time of diagnosis, the median value of random GH and IGF1 levels of all patients was 11.1 (IQR: 5.6; 25.7) μg/L and 731 (IQR: 491; 923) μg/L, respectively; 143 (64.2%) patients had a macroadenoma, whereas the remaining 80 (35.8%), had a microadenoma. A total of 176 patients (78.9%) underwent pituitary surgery, whereas radiation therapy or radiosurgery was used in 36 patients (16.2%). Medical therapy with somatostatin analogues (SRLs) and/or pegvisomant (PEG) was required in 98 and 67 patients, respectively. Table 2 shows the medical history of patients included in the current study. According to guidelines (2), acromegaly was considered to be controlled if mean fasting GH levels were not greater than 1 μg/L in the presence of normal IGF1 levels for age (only IGF1 levels for patients receiving PEG). According to IGF1 levels, patients were classified as uncontrolled (defined as IGF1 > 1.3 × upper limit of normal, ULN, *n* = 16) and controlled, either fully (defined as an IGF1 < 1 × ULN, *n* = 147) or partially (defined as IGF1 1.0–1.3 × ULN, *n* = 60). Comorbidities, including glucose abnormalities, dyslipidaemia, hypogonadism, arthralgia, osteoporosis, respiratory, and cardiovascular disease, were also investigated as adjunctive factors contributing to the psychological impact of acromegaly.

**Table 1:** Patient profile at study entry.

Profile characteristics	Values
Outpatient clinics, *n*	223
Naples	69 (30.9%)
Padua	62 (27.8%)
Messina	39 (17.5%)
Milan	31 (13.9%)
Pisa	22 (9.87%)
Age (years) at assessment*	56.0 (48.0, 64.5)
Sex	
Male	94 (42.2%)
Female	129 (57.8%)
Marital status	
No relationship	33 (14.8%)
Married	153 (68.6%)
Divorced	16 (7.17%)
Widowed	21 (9.42%)
Degree	
Primary school diploma	26 (11.7%)
Middle school diploma	77 (34.5%)
High school diploma	85 (38.1%)
University degree	35 (15.7%)

*Value presented as median (Q1, Q3).

**Table 2:** Medical history of patients included in the current study. Data are presented as median (Q1, Q3) or as *n* (%).

Characteristics	Values
Patients, *n*	223
Disease duration in years	5.00 (2.00; 10.0)
IGF1 value at diagnosis	731 (491; 923)
GH random value at diagnosis	11.1 (5.60; 25.7)
GH nadir post OGTT at diagnosis	8.8 (4.03; 17.3)
Adenoma size at diagnosis	
Microadenoma	80 (35.8%)
Macroadenoma	143 (64.2%)
Neurosurgery	
Transsphenoidal	167 (74.9%)
Transcranial	9 (4.04%)
None	47 (21.1%)
Hormonal deficit	
1	39 (17.6%)
2	16 (7.2%)
3	5 (2.2%)
Replacement therapy*	
No	177 (79.4%)
Yes	46 (20.6%)

*For hormonal deficiency at diagnosis.

GH, growth hormone; IGF1, insulin-like growth factor 1; OGTT, oral glucose tolerance test.

### Study protocol

A quantitative analysis involving the collection of data in numerical form was performed by using questionnaires to focus on when, where, what, and how often acromegaly influenced psychological morbidity. The first essential evaluation was a global psychological assessment, analysing depression and anxiety status, together with body image perception. Three self-report questionnaires were chosen to aim the goal: the Beck Depression Inventory-II (BDI-II) (27), the State-Trait Anxiety Inventory (STAI) Form Y 1 and 2 (28), and the body uneasiness test (BUT) A and B (29). The second evaluation was the sleep quality. Three different self-report questionnaires were chosen to reach an extensive analysis of every aspect of sleep dimension: the Berlin Questionnaire (30) aiming at exploring the experience of nocturnal sleep apnoeas; the Epworth Sleepiness Scale (31) focusing on diurnal sleepiness; and the Pittsburgh Sleep Quality Inventory (PSQI) (32) investigating the general quality of sleep. Considering the lack of sexual investigation in the acromegalic population, as the third evaluation, a wide sexual assessment was planned: the sexual function in men and women was assessed by the gender-related self-report questionnaires International Index for Erectile Function (IIEF)-15 (33) and Female Sexual Function Index (FSFI) (34), which aimed to collect an extensive representation of the relationship between acromegalic condition and sex experience. The fourth evaluation was the cognitive function, which was assessed by the following standardized procedures aimed at collecting data on visuospatial and verbal working memory, divided and selective attention, and verbal fluency: the Corsi Block-Tapping Task, forward and backward forms (35, 36, 37), Digit Span forward and backward forms (38), Trial Making Test (TMT) A and B (39, 40), and Phonemic Verbal Fluency – FAS (41). The last evaluation was the QoL, performed by the administration of the Acromegaly quality of life (AcroQoL, (42)), a specific questionnaire developed to measure the QoL in patients with acromegaly. In each centre, acromegalic patients have been assessed by a psychologist who administered the questionnaires in association with an interview. The study was approved by the ‘Federico II’ University Ethical Committee. This research complied with the Declaration of Helsinki. Written informed consent has been obtained from each patient after a full explanation of the purpose and nature of the entire cohort of procedures used in the study.

### Statistical analysis

Data were analysed using SPSS Software for Windows, version 27 (SPSS, Inc.). Data are reported as mean ± s.d., median, or IQR according to their distribution, unless otherwise specified. The comparison between the numerical data was made by one-way ANOVA, followed by the Bonferroni test for the adjustment of multiple comparisons. The comparison between controlled and uncontrolled patients, as well as between surgically and medically treated patients, was performed by independent-samples *t*-test. The comparison between prevalence was performed by *χ* ^2^ test corrected by Fisher exact test, when necessary. The correlation study was done by calculating Pearson’s correlation coefficients. Regression analysis was done to identify the best predictors of psychological impairment among GH at diagnosis (expression of baseline disease activity), IGF1 at evaluation (expression of disease control at the time of the study), age, sex, and disease duration. Significance was set at 5%.

## Results

The median age at study entry was 56 (IQR: 48; 64.5) years. The median estimated disease duration of acromegaly was 5 (IQR: 2; 10) years. At study entry, 38.1% of patients have reached a high school degree and 68.6% declared to have a couple relationship.

**Figure 1:**
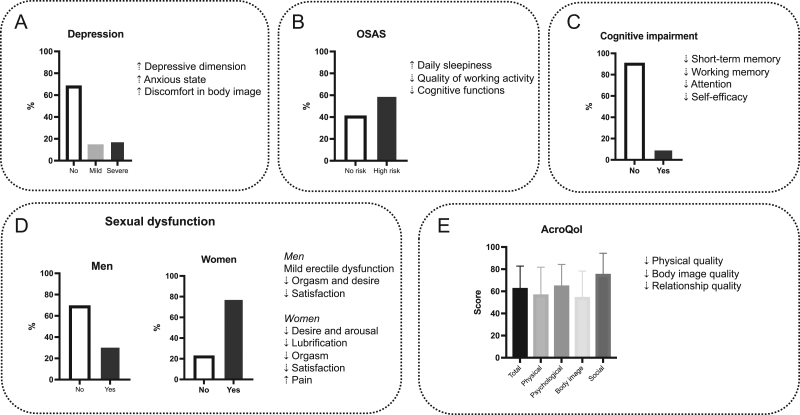
Psychological burden of acromegaly. Acromegaly inexorably causes body changes and loss of control on the body, resulting in a remarkable psychological impact, as body and mind are strictly interlinked, and many psychological factors (awareness of being ill, poor sleep quality and quality of life, compromised sexuality, and cognitive functions) seem to be crucial in the management of this chronic and debilitating disease. Therefore, acromegaly is associated with increased psychological morbidity. Global psychological health (A) is impaired mainly in the depressive dimension due to the increase in anxious state and discomfort in body image. Sleep quality (B) is also aggravated by increased daily sleepiness, reduced quality of working ability, and limited cognitive functions. Cognitive impairment (C) results in a remarkable reduction of working memory, as well as decreased attention and self-efficacy. In addition, sexual function (D) is compromised in both sexes, mainly because of a mild erectile dysfunction in men, reduced lubrification, and increased pain in women, with orgasm, desire, and satisfaction lowering in both sexes. Overall quality of life (E) is scant, particularly in the physical, body image, and relationship quality domains.

At study entry, the median values of random GH and IGF1 levels of the totality of patients were 1.16 (IQR: 0.4; 2.5) μg/L and 177 (IQR: 136; 234) μg/L, respectively; 168 (76.7%) patients had a macroadenoma, whereas the remaining 51 (23.3%) had a microadenoma. Of the 223 patients, 87 (39%) were treated with SRLs, 29 (13%) with PEG, 3 (1.3%) with cabergoline (CAB), and 46 (20.6%) with combined treatments, including 30 with SRL + PEG, 13 with SRL + CAB, and 3 with PEG+CAB, whereas 58 (26%) patients were not receiving medical therapy. At the time of the evaluation, among the 165 patients receiving medical therapy, 131 (79.4%) were controlled by medical therapy and 34 (20.6%) had active disease despite medical therapy. Median IGF1 levels and IGF1 × ULN at the study entry were 177 (IQR: 136; 234) µg/L and 0.848 (IQR: 0.642; 1.11), respectively. At study entry, the most common comorbidities were glucose abnormalities (50.2%), arterial hypertension (55.2%), arthralgia (55.9%), and dyslipidaemia (36.8%). Table 3 shows clinical and hormonal data at the study entry. The main results of the study are summarized in Fig. 1.

**Table 3:** Clinical and hormonal data at study entry. Data are presented as median (Q1; Q3) or as *n* (%).

Characteristics	Values
IGF1	177 (136; 234)
IGF1 × ULN	0.848 (0.642; 1.11)
≤1	147 (65.9%)
1.0–1.3	60 (26.9%)
>1.3	16 (7.7%)
GH random value at study entry^#^	1.16 (0.4; 2.5)
Disease activity*	
Disease control	131 (79.4%)
No disease control	34 (20.6%)
Treatment	
None	58 (26%)
Pegvisomant	29 (13%)
Somatostatin analogues	87 (39%)
Cabergoline	3 (1.4%)
Combined treatment	46 (20.6%)
Comorbidities at study entry	
Glucose abnormalities	
No	111 (49.8%)
Yes	112 (50.2%)
Impaired glucose tolerance	16 (7.2%)
Impaired fasting glucose	31 (13.9%)
Diabetes mellitus	65 (29.1%)
Dyslipidaemia	
No	141 (63.2%)
Yes	82 (36.8%)
Hypertension	
No	100 (44.8%)
Yes	123 (55.2%)
Heart disease	
No	139 (63.2%)
Yes	84 (36.8%)
Arrhythmia	
No	198 (88.8%)
Yes	25 (11.2%)
Arthralgia	
No	98 (44.1%)
Yes	125 (55.9%)
Osteoporosis	
No	186 (83.3%)
Yes	37 (16.7%)
Obstructive sleep apnoea syndrome	
No	189 (84.8%)
Yes	34 (15.2%)

*Calculated on 165 patients receiving medical therapy; ^#^Calculated on patients not receiving pegvisomant treatment.

GH, growth hormone; IGF1, insulin-like growth factor 1; ULN, upper limit of normal.

### Global psychological assessment

The BDI-II total score (BDI-II questionnaire, Supplementary Table 1, see section on supplementary materials given at the end of this article), assessing the typical syndrome of depression including somatic-affective symptoms, such as pessimism and irritability, and cognitive symptoms, such as guilt or feelings of being punished, revealed that the average settled on 65.9 ± 25.3 percentile (normal score < 85 percentile) in the entire patient population. In particular, in 68.2% of the patient cohort, a depression condition did not represent a significant pathologic dimension, whereas the remaining 31.8% spreads out in the categories highlighting a depression syndrome, which was mild (score 85–90 percentile) in 9.9%, moderate (score 91–95 percentile) in 4.5%, and severe (score > 95 percentile) in 17.5% of cases. The average score for depression was significantly higher in patients younger than 45 years (74.5 ± 21.2) as compared to those aged 45–64 (64.3 ± 25.8, *P* = 0.047) and > 64 (63.0 ± 26.1, *P* = 0.05) years. A depressive condition was indeed slightly more prevalent in patients younger than 45 years, in whom it was registered in 40.4% and categorized as severe in 23.8% of cases, compared to those aged 45–64 or >64 years, where a depressive syndrome was absent in 68.8 and 73.2%, respectively. No relevant difference was found in the average score and prevalence (37.2 vs 27.9%) of depressive syndrome between men and women, with a slight predominance in men, in whom a severe degree was registered in 19.1 vs 16.3% of women. The average score of depression was slightly higher in patients with a disease duration less than 2 years as compared to the different groups of disease durations; the depressive condition was also slightly more prevalent in patients with a disease duration less than 2 years, in whom it was registered in 48%, and categorized as severe in 28% of cases, compared to the patients with disease duration of 2–5 years, 5–10 years, or more than 10 years, where a depressive condition was absent in 63.6, 77.9, and 67.3% of patients, respectively.

A similar distribution has been found for the BDI-II questionnaire sub-scores relative to somatic-affective and cognitive domains. In particular, the total score revealed that the average settled on 66.6 for the somatic-affective scale (normal score < 85 percentile) and 71.7 for the cognitive scale (normal score < 85 percentile) in the entire patient population. Overall, a somatic-affective mood lowering, as a contributor to depression, was found in 30% of patients, resulting mild (score 85–90 percentile) in 8.5%, moderate (score 91–95 percentile) in 6.3%, and severe (score > 95 percentile) in 15.2% of cases. The average score for somatic-affective mood lowering was significantly higher in patients younger than 45 years (75.2 ± 19.8) as compared to those aged 45–64 (65.9 ± 24.5, *P* = 0.021) and > 64 (61.7 ± 25.6, *P* = 0.009) years. A somatic-affective mood lowering was slightly more prevalent in patients younger than 45 years, in whom it was registered in 38% and categorized as severe in 21.4% of cases, compared to those aged 45–64 years or >64 years, where a somatic-affective mood lowering was absent in 70.4 and 75%, respectively. No relevant difference was found in the average score, and prevalence (36.1 vs 25.6%), of somatic-affective mood lowering between men and women, with a slight predominance in men, in whom a severe degree was registered in 18.1 vs 13.2% of women. The average scores of somatic-affective mood lowering were slightly higher in patients with a disease duration less than 2 years as compared to the different groups of disease durations; the somatic-affective mood lowering was slightly more prevalent in patients with disease duration less than 2 years, in whom it was registered in 48%, and categorized as severe in 24% of cases, compared to the patients with disease duration of 2–5 years, 5–10 years, or more than 10 years, where a somatic-affective mood lowering was even absent in 69.1, 71.1, and 74.9% of patients, respectively. A cognitive impairment, considered as contributor of depression, mainly related to self-dislike and worthlessness that reflect negative views of self, and/or past failure and pessimism that reflect global negative views, was found in 39.5% of patients and categorized as mild (score: 85–90 percentile) in 15.2%, moderate (score: 91–95 percentile) in 6.7%, and severe (score: > 95 percentile) in 17.4% of patients. No relevant difference was found in the average scores for cognitive symptoms among age groups. A cognitive impairment was slightly more prevalent in patients younger than 45 years, in whom it was registered in 45.2% and categorized as severe in 21.4% of cases, compared to those aged 45–64 years or >64 years, where cognitive symptoms were absent in 63.1 and 58.9%, respectively. No relevant difference was found in the average scores and prevalence (43.6 vs 36.4%) of cognitive symptoms between men and women, with a slight predominance in men, considering all the degrees, but a slight predomince of severe degree in women, in whom it was registered in 20.2 vs 13.8% of men. The average scores of cognitive symptoms were slightly higher in patients with disease duration less than 2 years as compared to the different groups of disease durations; the cognitive symptoms were slightly more prevalent in patients with disease duration less than 2 years, in whom it was registered in 52%, and categorized as severe in 16% of cases, compared to the patients with disease duration of 2–5 years, 5–10 years, or more than 10 years, where cognitive symptoms were absent in 58.2, 69.4, and 53.9% of patients, respectively.

When considering patients receiving medical therapy, no relevant difference was seen in average scores for depression, as well as somatic-affective mood lowering and cognitive impairment, between patients with controlled and uncontrolled disease. Depression syndrome and cognitive impairment were similar or slightly less prevalent in patients with controlled (30.8 and 39.1%, respectively) compared to those with uncontrolled (34.3 and 40.3%, respectively) disease. Conversely, a significant difference (*P* = 0.037) was registered in the prevalence of somatic-affective mood-lowering between patients with controlled (27.5%) and uncontrolled (35.8%) disease. When patients were stratified according to their IGF1 × ULN, average scores, as well as prevelance for depression syndrome, somatic-affective mood lowering, and cognitive impairment were slightly higher in patients with partial disease control as compared to patients with full disease control and uncontrolled disease. No significant difference was found in the average scores and prevalence of depression syndrome, somatic-affective mood lowering, and cognitive impairment among patients with 0–1, 2–4, or more than 4 comorbidities.

The STAI Form Y (Supplementary Table 2) assesses the person’s subjective perception of the present dimensions correlated with anxiety. The STAI Form Y 1 (STAI Y 1) explores anxiety state. For the STAI Y 1, the average score for the whole patient cohort was 48.7 ± 11.8 (normal score ≤ 40). Overall, 82.3% declared discomfort about the anxiety dimension (uneasiness, tension, upset, fear, agitation, confusion), which was mild (score 40–50) in 42.7%, moderate (score 51–60) in 25%, and severe (score > 60) in 14.6%. The average score for state anxiety was significantly higher in patients younger than 45 years (54.9 ± 11.9) as compared to those aged 45–64 (47.5 ± 11.4, *P* < 0.001) and > 64 (46.6 ± 11.4, *P* = 0.002) years. The prevalence of the severe form of the state anxiety was significantly higher in patients younger than 45 years (54.8%), compared to those aged 45–64 years (28.8%) or >64 years (30.4%), whereas the mild form was significantly higher patients older than 64 years (41.1%) compared to those aged < 45 years (21.4%) or 45–64 years (39.2%; *P* = 0.033). No relevant difference was found in the average score and prevalence of state anxiety between men and women (68.1 vs 60.4%), with the severe form being slightly more predominant in men (39.4 vs 30.2%) and the mild form or absence, slightly more prevalent in women (39.5% vs 31.9%). No relevant difference was found in the average scores for state anxiety among different disease durations. The state anxiety was slightly more prevalent in those patients with disease duration less than 2 years, in whom it was registered in 68%, and categorized as severe in 36% of cases, compared to the patients with disease duration of 2–5 years, 5–10 years, or more than 10 years where a state anxiety was absent in 34.5, 39 and 46.2% of patients, respectively.

The STAI Form Y 2 (STAI Y 2, Supplementary Table 2) explores trait anxiety and assesses the general trend to perceive situations and face them with a stable anxious mood. For the STAI Y 2, the average score in the whole cohort was 51.6 ± 12.8 (normal score ≤ 40). Overall, 80.4% declared to feel discomfort and anxiety when facing stressful situations, which was mild (score 40–50) in 32.3%, moderate (score 51–60) in 26.5%, and severe (score > 60) in 21.5%. The average score for trait anxiety was significantly higher in patients younger than 45 years (56.8 ± 12.3) as compared to those aged 45–64 (50.6 ± 12.1, *P* = 0.0013) and > 64 (49.9 ± 13.9, *P* < 0.001) years. Trait anxiety was slightly more prevalent in patients younger than 45 years, particularly for the severe form which was registered in 54.8% of cases, compared to those aged 45–64 years or >64 years (31.2 and 41.1%, respectively). No relevant difference was found in the average scores, and prevalence (68.1 vs 69%) of trait anxiety between men and women. No significant difference was found in the average scores for trait anxiety among different disease durations. The trait anxiety was slightly more prevalent in those patients with disease duration less than 2 years, in whom it was registered in 76%, and categorized as severe in 48% of cases, compared to the patients with disease duration of 2 –5 years, 5–10 years, or more than 10 years where a trait anxiety was absent in 25.5, 28.8, and 34.6% of patients, respectively.

At both STAI Y 1 and STAI Y 2 tests, no relevant difference was found in the average scores and prevalence of state and trait anxiety between patients with controlled and those with uncontrolled disease. When considering patients receiving medical therapy, in patients with controlled disease, prevalence of state (66%) and trait (70.5%) anxiety was slightly higher as compared to those with uncontrolled disease (58.3 and 64.2%, respectively). When patients were stratified according to their IGF1 × ULN, no relevant difference was found in the average scores for state and trait anxiety among patients with full disease control, partial disease control, and uncontrolled disease. State and trait anxiety was slightly more prevalent in patients with partial disease control (68.8 and 71.9%, respectively) compared to those with full disease control (66 and 70.5%, respectively) and those with uncontrolled disease (48.6 and 57.1%, respectively). No relevant difference was found in the average scores and prevalence of state and trait anxiety between patients with 0–1, 2–4, or more than 4 comorbidities.

The BUT-A (Supplementary Table 3) revealed an average score in the whole cohort of 0.8 ± 0.9 (Global Severity Index score normal value ≤ 1.2). This test, which measures weight phobia, body image concerns, avoidance, compulsive self-monitoring, detachment, and estrangement feelings towards one’s own body (depersonalization), revealed that 21.3% of the patient cohort experienced global concerns about body image. Average scores for global BUT-A were significantly higher in patients younger than 45 years (1.4 ± 1.0) as compared to those aged 45–64 years (0.8 ± 0.8, *P* < 0.001) and >64 years (0.4 ± 0.4, *P* < 0.001). Discomfort in body image was significantly more prevalent (*P* < 0.001) in patients younger than 45 years (47.6%) compared to those aged 45–64 (18.5%) and >64 years (7.3%), as well as in patients aged 45–64 years compared to those aged > 64 years (*P* < 0.001). Average scores for global BUT-A (*P* = 0.004) were significantly higher in women (0.9 ± 0.1) as compared to men (0.6 ± 0.6). No relevant difference was seen in the prevalence of global concerns about body image between men (16.1%) and women (25%), although a slight predominance was observed in women. Average scores for global BUT-A were slightly higher in patients with disease duration less than 2 years as compared to other disease durations. Discomfort in body image was slightly more prevalent in those patients with disease duration less than 2 years, in whom it was registered in 36%, compared to the patients with disease duration of 2–5 years, 5–10 years, or more than 10 years where discomfort in body image was absent in 76.4, 87.7, and 82.7% of patients, respectively.

Regarding BUT-A subscales, the overall prevalence of weight phobia, body image concerns, avoidance, compulsive self-monitoring, and depersonalization were 32.6, 37.1, 14.9, 28.1, and 14%, respectively. Average scores for weight phobia (*P* < 0.001), body image concerns (*P* < 0.001), avoidance (*P* = 0.002), compulsive self-monitoring (*P* < 0.001), and depersonalization (*P* < 0.001) were significantly higher in patients younger than 45 years (1.7 ± 1.3, 1.8 ± 1.2, 0.8 ± 0.9, 1.3 ± 1.0, and 0.9 ± 1.1, respectively) as compared to those aged 45–64 years (1.0 ± 1.1, 1.0 ± 1.0, 0.4 ± 0.7, 0.7 ± 0.8, and 0.4 ± 0.9, respectively) and >64 years (0.4 ± 0.6, 0.6 ± 0.6, 0.3 ± 0.5, 0.3 ± 0.4, and 0.1 ± 0.3, respectively). Weight phobia (*P* < 0.001), body image concerns (*P* < 0.001), compulsive self-monitoring (*P* < 0.001), and depersonalization (*P* < 0.001) were significantly more prevalent in patients younger than 45 years (61.9, 64.3, 54.8, and 33.3%, respectively) as compared to those aged 45–64 years (32.3, 36.3, 27.4, and 12.1%, respectively) and >64 years (10.9, 18.2, 9.1, and 3.6%, respectively). Similarly, weight phobia (*P* = 0.003), body image concerns (*P* = 0.002), and compulsive self-monitoring (*P* = 0.006) were significantly more prevalent in patients aged 45–64 years as compared to those aged > 64 years. Average scores for weight phobia (*P* < 0.001), body image concerns (*P* = 0.006), avoidance (*P* = 0.038), compulsive self-monitoring (*P* = 0.008), and depersonalization (*P* = 0.024) were significantly higher in women (1.2 ± 1.2, 1.2 ± 1.1, 0.6 ± 0.8, 0.9 ± 0.9, and 0.9 ± 1.0, respectively) as compared to men (0.7 ± 0.8, 0.8 ± 0.9, 0.4 ± 0.6, 0.6 ± 0.7, and 0.3 ± 0.6, respectively). No relevant difference was seen between men and women in the prevalence of weight phobia (28 vs 35.9%), body image concerns (31.2 vs 41.4%), avoidance (12.9 vs 16.4%) compulsive self-monitoring (23.7 vs 31.3%), and depersonalization (9.7 vs 17.2%), although with an apparent predominance of the single pathological domains in women. Average scores for weight phobia, body image concerns, and depersonalization were slightly higher in patients with disease duration less than 2 years, and that of avoidance in patients with disease duration of 5–10 years as compared to other disease durations, with no significant difference being seen in average scores for compulsive self-monitoring among different disease durations. For all subscales, the discomfort in body image was slightly more prevalent in those patients with disease duration less than 2 years, in whom it was registered in 44, 44, 20, 48, and 24%, respectively, compared to the patients with disease duration of 2–5 years, 5–10 years, or more than 10 years where weight phobia (63.6, 70.2, and 75%, respectively), body image concerns (54.5, 71.9, and 65.4%, respectively), avoidance (83.6, 87.7, and 86.5%, respectively), compulsive self-monitoring (72.7, 82.5, and 71.2%, respectively), and depersonalization (87.3, 91.2, and 84.6%, respectively) were absent.

The BUT-B (Supplementary Table 3) revealed an average score in the whole cohort of 2.1 ± 1.2 (Positive Symptom Distress Index, PSDI, normal value <1 ). This test, looking at specific worries about body parts or functions, revealed a moderate body image discomfort related to specific areas in the whole patient population. The body areas belong to eight general categories whose scores are normal if <1. The patients reported a high level of discomfort relative to specific areas: face shape (average score 1.2), nose (average score 1.9), teeth (average score 1.2), hands (average score 1.6), belly (average score 1.2), ankles (average score 1.5), and general body sounds (average score 1.2). For three categories, average scores were near to clinical cut-off: category I (mouth) score 0.9 ± 1.0; category III (thighs) score 0.8 ± 1.2; category IV (legs) score 1.0 ± 1.1. PSDI average score was significantly higher in patients younger than 45 years (2.6 ± 1.2) as compared to those aged 45–64 years (2.1 ± 1.2, *P* = 0.017) and >64 years (1.9 ± 1.1, *P* = 0.047). PSDI average score was significantly higher (*P* < 0.001) in women (2.4 ± 1.1) as compared to men (1.9 ± 1.2). PSDI average score was similar among patients with disease duration <2 years, 2–5 years, 5–10 years, and more than 10 years.

When considering patients receiving medical therapy, no relevant difference was found in average scores and prevalence of global concerns, weight phobia, body concerns, avoidance, compulsive self-monitoring, and depersonalization among patients with controlled disease as compared to those with uncontrolled disease. To a similar extent, PSDI score was only slightly higher in patients with uncontrolled disease as compared to those with controlled disease. When patients were stratified according to their IGF1 × ULN, no relevant difference was found in global and all subscales average scores among patients with full disease control as compared to those with partial disease control and uncontrolled disease. Similarly, no relevant difference was found in PSDI scores among patients with full disease control, partial disease control, and uncontrolled disease. To a similar extent, global concerns, weight phobia, body concerns, avoidance, compulsive self-monitoring, and depersonalization were slightly more prevalent in patients with uncontrolled disease as compared to those with full disease control and partial disease control. In patients with concomitant OSAS, average scores for BUT-A global score (*P* = 0.029), compulsive self-monitoring (*P* = 0.015), and depersonalization (*P* < 0.001) were significantly lower (0.5 ± 0.6, 0.5 ± 0.6, and 0.2 ± 0.4, respectively) as compared to patients without OSAS (0.8 ± 0.9, 0.8 ± 0.9, and 0.5 ± 0.9, respectively). Body image concerns were significantly less prevalent in patients with more than 4 comorbidities (18.6%) compared to those with 0–1 comorbidities (47.7%, *P* = 0.002) and those with 2–4 comorbidities (38.1%, *P* = 0.02). Similarly, compulsive self-monitoring was significantly less prevalent in patients with more than 4 comorbidities (14%) compared to those with 0–1 comorbidities (44.6%, *P* < 0.001) and those with 2–4 comorbidities (23.9%, *P* = 0.007), whereas comorbidities had no significant impact on the prevalence of weight phobia, avoidance, and depersonalization.

### Sleep quality

The Berlin Questionnaire (Supplementary Table 4), adopted to assess the risk of OSAS (normal score ≤ 1), highlighted that 59.2% of the patient population were at high risk, compared to the remaining 40.8%, displaying a low or absent risk to develop OSAS. OSAS risk was slightly more prevalent in patients aged 45–64 years (60.8%) and >64 years (60.7%) compared to those aged < 45 years, where OSAS risk was low or absent in 47.6% of cases. A slight predominance of high OSAS risk was found in men compared to women (63.8 vs 55.8%). High OSAS risk was slightly more prevalent in those patients with disease duration of 2–5 years or more than 10 years, in whom it was registered in 67.3 and 65.4%, respectively, compared to the patients with disease duration of 5–10 years (52.5%), and especially those with disease duration less than 2 years (44%).

The Epworth Sleepiness Scale (Supplementary Table 4), adopted to assess daily sleepiness (normal score: ≤ 10), showed that 19.3% were at risk of daily sleepiness, which was mild (score: 11–12) in 12.9%, moderate (score: 13–15) in 6.4%, and severe in 0% (score: 16–24) of cases. Daily sleepiness was similarly prevalent in patients aged < 45 years (19%), 45–64 years (20%) and > 64 years (17.9%). No relevant difference was found in the daily sleepiness between men and women (22.3 vs 17.1%), although with a slight predominance in men. Daily sleepiness was slightly more prevalent in patients with disease duration of 2–5 years (27.3%) compared to those with disease duration less than 2 years (16%), 5–10 years (16.9%) and more than 10 years (19.2%).

The PSQI (Supplementary Table 4), adopted to assess the overall sleep quality (normal score ≤ 5), revealed that 61% of the patients suffered from sleep disorders. Sleep disorders were slightly more prevalent in patients aged > 64 years (66.1%) compared to those aged < 45 years (54.8%) and 45–64 years (60.8%). No relevant difference was found in the daily sleepiness between men and women (57.4 vs 63.6%), although with a slight predominance in women. Sleep disorders were slightly more prevalent in patients with disease duration of 2–5 years (63.6%) and 5–10 years (61%) compared to those with disease duration less than 2 years (52%) and more than 10 years (57.7%).

When considering patients receiving medical therapy, no relevant difference in OSAS risk, daily sleepiness and sleep disorders was found between patients with controlled (59, 19.2, and 63.5%, respectively) and uncontrolled disease (59.7, 19.4, and 55.2%, respectively). When patients were stratified according to their IGF1 × ULN, patients with full disease control did not show a significant difference in OSAS risk, daily sleepiness, and sleep disorders (59, 19.2, and 63.5%, respectively) compared to patients with partial disease control (65.6, 21.9, and 59.4%, respectively) and those with uncontrolled disease (54.3, 17.1, and 51.4%, respectively). OSAS risk was significantly more prevalent in patients with more than 4 comorbidities (84.1%) as compared to those with 0–1 comorbidities (44.6%, *P* < 0.001) and those with 2–4 comorbidities (57.9%, *P* = 0.003), whereas the number of comorbidities were found not to affect daily sleepiness and sleep quality.

### Sexual function

The sexual function was assessed employing the gender-related self-report questionnaires IIEF-15 (Supplementary Table 5A) and FSFI (Supplementary Table 5B).

In men (*n* = 94, 54.8% eugonadal and 45.2% hypogonadal), the total IIEF-15 score, representing general sexual function during the last month, was 45.6 ± 23.8 on average (score: 5–75), with the erectile function subdomain score of 18.4 ± 11.1 on average (score: 1–30; normal score > 26). In terms of general satisfaction, 46.2% of men declared to be very satisfied and 23% moderately satisfied in the past 4 weeks. Conversely, 59.6% of men reported erectile dysfunction, described as mild (score 17–25) in 22.3%, moderate (score: 11–16) in 12.8%, and severe (score: 6–10) in 24.5%. Average scores for global IIEF (25.6 ± 26.3, *P* < 0.001), as well as for erectile dysfunction (9.5 ± 11.4, *P* < 0.001), orgasm (3.3 ± 4.7, *P* < 0.001), desire (3.5 ± 3.9, *P* < 0.001), and general satisfaction (4.0 ± 3.3, *P* < 0.001) were significantly lower in patients aged >64 years as compared to those aged < 45 years (51.1 ± 20.5, 21.6 ± 9.1, 7.3 ± 4.0, 7.6 ± 2.8, and 7.2 ± 2.7) and those aged 45–64 years (50.5 ± 20.3, 20.5 ± 10.1, 7.9 ± 3.9, 7.2 ± 2.9, and 6.8 ± 2.6), whereas no significant difference was found in total and subdomain scores between patients aged <45 years and those aged 45–64 years. The presence of erectile dysfunction was significantly more prevalent (*P*=0.021) in patients aged >64 years, in whom it was registered in 70.8% and categorized as severe in 62.5% of cases, compared to those aged < 45 and 45–64 years, where erectile dysfunction was absent in 61.1% and 48.1%, respectively. Average scores for general satisfaction and erectile function were slightly lower in patients with disease duration less than 2 years as compared to those with disease duration of 2–5 years, 5–10 years, and more than 10 years. No relevant difference was found in the prevalence of erectile dysfunction between the different groups of disease duration. The prevalence of erectile dysfunction was slightly higher in hypogonadal (52.4%) than in eugonadal (43.1%) patients.

When considering patients receiving medical therapy, average scores for global IIEF, erectile function, and general satisfaction were slightly higher, whereas those of orgasm and desire were slightly lower in patients with disease control as compared to those with uncontrolled disease. Erectile dysfunction was significantly (*P* = 0.006) less prevalent in patients with controlled disease (52.8%) compared to those with uncontrolled disease (87.5%). When patients were stratified according to their IGF1 × ULN, average scores for global IIEF, erectile function, orgasm, desire, and general satisfaction were slightly lower in patients with uncontrolled disease as compared to those with full or partial disease control. Erectile dysfunction was slightly less prevalent in patients with full disease control (52.8%) compared to patients with partial disease control (75%) and those with uncontrolled disease (66.7%). Prevalence of erectile dysfunction progressively increased from patients with 0–1 (60.4%) to those with 2–4 (62.5%) or more than 4 comorbidities (88.9%). Particularly, in men with cardiovascular disease, the prevalence of erectile dysfunction was significantly higher (*P* = 0.033) than in those without cardiovascular disease (38.5 vs 17.3%).

In women (n = 129, 89% eugonadal and 11% hypogonadal), the total FSFI score, representing the general sexual function, was 15.7 ± 11.3 on average (normal value ≥ 26.55); 77.5% of the women experienced sexual dysfunction, whereas 22.5% of women reported a normal sexual function. Average scores for global FSFI (8.0 ± 8.5, *P* < 0.001), as well as for desire (2.3 ± 1.5, *P* = 0.034), arousal (1.0 ± 1.5, *P* < 0.001), lubrification (0.8 ± 1.2, *P* < 0.001), orgasm (0.8 ± 1.2, *P* < 0.001), general satisfaction (2.1 ± 1.6, *P* = 0.004), and pain (1.4 ± 2.4, *P* = 0.012) were significantly lower in patients aged > 64 years old as compared to those aged <45 years (20.0 ± 11.6, 3.4 ± 1.3, 2.9 ± 1.9, 3.5 ± 2.3, 3.5 ± 2.3, 3.8 ± 1.7, and 3.5 ± 2.4, respectively) and those aged 45–64 years (17.0 ± 10.7, 3.1 ± 1.6, 2.6 ± 2.0, 2.9 ± 2.3, 2.7 ± 2.2, 3.0 ± 1.8, and 2.8 ± 2.5, respectively). Sexual dysfunction was significantly more prevalent in patients aged > 64 years (95.8%) as compared to those aged <45 years (60%, *P* = 0.011) and slightly more prevalent than in those aged 45–64 years (77.4%). Average scores for desire were significantly higher (*P* = 0.05) in patients with disease duration less than 2 years (3.7 ± 1.4) as compared to those with disease duration of 2–5 years (3.0 ± 1.7), 5–10 years (3.6 ± 1.8), and more than 10 years (2.5±1.2), whereas average scores for global FSFI, as well as for arousal, lubrification, orgasm, general satisfaction, and pain were similar among the different disease duration groups. Overall, sexual dysfunction was slightly more prevalent in patients with disease duration more than 10 years (85.7%) compared to those with disease duration less than 2 (80%), 2–5 (77.8%), and 5–10 years (74.1%). The prevalence of sexual dysfunction was similar in hypogonadal and eugonadal patients (75 vs 77.6%).

When considering patients receiving medical therapy, average scores for desire were significantly higher (*P* = 0.011) in patients with controlled (3.3 ± 1.6) as compared to those with uncontrolled (2.5 ± 1.4) disease, whereas global FSFI and the remaining different subdomain scores were not relevantly different in patients with uncontrolled and those with controlled disease. Sexual dysfunction was slightly less prevalent in patients with controlled compared to those with uncontrolled disease (76.7 vs 78.9%). When patients were stratified according to their IGF1 × ULN, average scores for desire were significantly lower (*P* = 0.044) in patients with uncontrolled disease (2.7 ± 1.3) compared to those with full (3.3 ± 1.6) or partial (2.4 ± 1.5) disease control. Sexual dysfunction was slightly less prevalent in patients with full disease control (76.7%) and patients with partial disease control (77.8%) compared to those with uncontrolled disease (80%). The prevalence of sexual dysfunction significantly (*P* = 0.009) increased from women with 0–1 comorbidities (61.1%) to those with 2–4 comorbidities (82.1%) and with more than 4 comorbidities (94.7%). Particularly, in women with cardiovascular disease, the prevalence of sexual dysfunction was significantly higher (*P* = 0.014) than in those without this complication (93.9 vs 70.5%).

### Cognitive functions

Cognitive tests, such as the Corsi Block-Tapping Task (forward and backward forms), the Digit Span (forward and backward forms), the TMT (TMT-A, TMT-B, and TMT B-A), and Phonemic Verbal Fluency – FAS, were used to investigate the main cognitive functions, including visuospatial and verbal working memory, divided and selective attention, and verbal fluency (Supplementary Table 6).

The Corsi Block-Tapping Task and the Digit Span in the forward and backward forms were used to assess visuospatial and verbal short-term working memory. The average scores for the whole patient cohort for visuospatial and verbal forward working memory were 4.8 ± 1.3 (normal score > 1) and 6.0 ± 1.5 (normal score > 1) whereas the avarage scores for the whole patient cohort for visuospatial and verbal backward working memory were 3.8 ± 1.4 (normal score >1) and 3.9 ± 1.3 (normal score >1). According to the forward forms of the tests, overall visuospatial and verbal working memory troubles were found in 9.5 and 14.5% of patients, whereas according to the backward forms, memory troubles were found in 9.5 and 12.7% respectively. Average scores for the Corsi Block-Tapping Task and Digit Span forward were slightly higher in patients aged < 45 years as compared to those aged 45–64 years and those aged > 64 years. Visuospatial and verbal working memory troubles were slightly more prevalent in patients aged 45–64 years compared to those aged < 45 years and > 64 years. Average scores for Corsi Block-Tapping Task backward form were significantly higher (*P* = 0.028) in men (4.1 ± 1.4) compared to women (3.6 ± 1.3), whereas average scores for Corsi Block-Tapping Task forward and Digit Span forward and backward were similar in men and women, slightly higher in men compared to women, and almost significantly (*P* = 0.051) higher in men compared to women, respectively. Visuospatial and verbal working memory troubles were slightly more prevalent in women compared to men, with the exception of troubles in verbal working memory forward, which seems to be slightly more prevalent in men than in women. Average scores for the Corsi Block-Tapping Task and Digit Span were similar in patients of the different disease duration groups. Visuospatial and verbal working memory troubles were slightly more prevalent in patients with disease duration of 5–10 years or more than 10 years compared to those with disease duration less than 2 years or 2–5 years.

The TMT in its three different parts (TMT-A, TMT-B, and TMT B-A) was used to assess divided and selective attention. Scoring for all TMT tests is based on number of seconds required to complete the task; patients are scored according to each part of the TMT tests and higher scores reveal greater impairment. The average score for the whole patient cohort was 33.9 ± 17.3 for TMT-A, 90.7 ± 69.5 for TMT-B, and 57.4 ± 63.8 for TMT B-A. Overall, the attentive process was reduced in 9.7–12.3% of patients. Average scores for the TMT-A, TMT-B, and TMT B-A were slightly lower in patients aged 45–64 years as compared to those aged < 45 years and >64 years. The reduced attentive process was slightly more prevalent in patients aged > 64 years as compared to those aged < 45 years and 45–64 years. Average scores for TMT-B were almost significantly higher (*P* = 0.05) in women (98.5 ± 74.7) compared to men (80.3 ± 60.8), whereas those of TMT-A and TMT B-A are slightly higher in women (35.4 ± 17 and 63.5 ± 69.8) than in men (31.9 ± 17.6 and 49.1 ± 54.0). No relevant difference between men and women was seen in the prevalence of the reduced attentive process (7.5 vs 15.7, 5.4 vs 12.9 and 6.4 vs 12.1%), although a slight predominance was registered in women. Average scores for the TMT-A were slightly higher in patients with disease duration of 2–5 years as compared to those with disease duration less than 2 years, 5-10 years, and more than 10 years. TMT-B and TMT B-A scores were slightly lower in patients with disease duration less than 2 years as compared to those with disease duration of 2–5 years, 5–10 years, and more than 10 years. The reduced attentive process was slightly more prevalent in patients with disease duration of 2–5 years or more than 10 years as compared to those with disease duration less than 2 years, and of 5–10 years.

Lastly, the Phonemic Verbal Fluency – FAS was used to assess the verbal fluency, as expression of frontal executive functions. The average score for the whole patient cohort was 30.7 ± 9.3 (normal score > 17). Overall, verbal fluency troubles were found in 11.5% of the whole patient cohort. Average scores for the FAS were slightly higher in patients aged 45–64 years as compared to those aged < 45 years and those aged > 64 years. Verbal fluency troubles were slightly more prevalent in patients aged < 45 years as compared to those aged 45–64 years and >64 years. Average scores for FAS and prevalence of verbal fluency troubles were similar in men and women. Average scores for the FAS were slightly lower in patients with disease duration more than 10 years as compared to those with disease duration less than 2 years, 2–5 years, and 5–10 years. Verbal fluency troubles were slightly more prevalent in patients with disease duration more than 10 years (16%) as compared to those with disease duration < 2 years, 2–5 years, and 5–10 years, in whom verbal fluency was normal in 96, 92.7, and 89.3%, respectively.

When considering patients receiving medical therapy, no relevant difference was registered in cognitive function questionnaire scores between patients with controlled and those with uncontrolled disease. Visuospatial and verbal working memory, attentive process, and verbal fluency troubles were not relevantly different between patients with controlled and those with uncontrolled disease. When patients were stratified according to their IGF1 × ULN, average scores for Corsi Block-Tapping Task, Digit Span, and FAS were not relevantly different among patients with full or partial disease control and uncontrolled disease, with average scores for TMT-A, TMT-B and TMT B-A slightly higher in patients with uncontrolled disease or partial disease control compared to patients with full disease control. The prevalence of visuospatial and verbal working memory, attentive process, and verbal fluency troubles were not relevantly different among patients with full or partial disease control and uncontrolled disease, with verbal fluency troubles slightly more prevalent in patients with uncontrolled disease. No relevant difference was found in cognitive function questionnaire scores between patients with 0–1, 2–4, or more than 4 comorbidities. Nevertheless, the TMT-BA score was significantly lower (*P* < 0.001) in patients with concomitant OSAS (37 ± 28.6) as compared to those without this comorbidity (61.1 ± 67.7).

### Quality of life

The AcroQoL (Supplementary Table 7), a self-administered disease-specific questionnaire to measure the QoL in patients with acromegaly (22 questions with 5 possible responses scored 1–5, high score good with 100 indicating the best possible QoL on a scale from 0 to 100), revealed an average score of 62.4 ± 20.4. AcroQoL average scores were slightly lower in patients aged < 45 years and in those aged 45–64 years as compared to those aged > 64 years. No relevant difference was found in AcroQoL average scores between men and women (64.1 ± 21.8 vs 61.2 ± 19.2), although with a slightly higher score in men. Global scores of AcroQoL were slightly lower in patients with disease duration less than 2 years and those with disease duration of 2–5 years as compared to those with disease duration of 5–10 years and more than 10 years. Among AcroQoL subscales, no significant difference was found in AcroQoL subscale average scores across different patient ages, whereas average scores for social relationships were significantly higher in patients > 64 years (80.8 ± 15.1) as compared to those aged < 45 years (72.0 ± 19.3, (*P* = 0.022)) and those aged 45–64 years (73.8 ± 20.6, *P* = 0.05). No relevant difference was found in AcroQoL subscale average scores between men and women. Average scores of physical, psychological, body image, and social relationship quality subscales were similar across different disease durations.

When considering patients receiving medical therapy, global AcroQoL average score was slightly higher in patients with controlled as compared to those with uncontrolled disease. Similarly, average scores for physical, psychological, body image, and social relationship quality subscales were slightly higher in patients with controlled disease than those with uncontrolled disease. When patients were stratified according to their IGF1 × ULN, average score for body image quality was significantly lower in patients with partial disease control (average score 44.7 ± 23.6) as compared to those with full disease (average score 55.1 ± 23.8, *P* = 0.039) and those with an uncontrolled disease (average score 59.3 ± 23.7, *P* = 0.02), as well as that of social relationship significantly lower in patients in patients with partial disease control (70.7 ± 20.9) compared to those with uncontrolled disease (78.6 ± 17.6, *P* = 0.027), whereas no relevant difference was found in the average global AcroQoL and other subscales scores. No relevant difference was found in AcroQoL scores among patients with 0–1, 2–4, or more than 4 comorbidities; however, physical quality average score was significantly lower (*P* < 0.001) in patients with concomitant arthralgia and osteoporosis (51 ± 24.1) as compared to those not affected by these comorbidities (64.4 ± 25.1).

### Correlation study

Correlations of five clinical parameters, including patient age, disease duration, GH at diagnosis, IGF1 at evaluation, and IGF1 × ULN, with psychological findings were investigated. Patient age inversely correlated with BDI total score (r = −0.189, *P* =  0.002) and relative domains of somatic-affective (r = −0.245, *P* < 0.001) and cognitive (r = −0.335, *P* < 0.001) scales, STAI Y 1 (r = −0.228, *P* < 0.001) and STAI Y 2 (r = −0.163, *P* = 0.008) scores, BUT-A total (r = −0.379, *P* < 0.001) and weight phobia (r = −0.388, *P* < 0.001), body image (r = −0.366, *P* < 0.001), avoidance (r = −0.232, *P* < 0.001), compulsive self-monitoring (r = −0.346, *P* < 0.001), and depersonalization (r = −0.286, *P* < 0.001) total scores, PSDI total score (r = −0.131, *P* = 0.035), as well as with PSQI sleep (r = −0.210, *P* < 0.001), total FSFI score (r = −0.186, *P* =  0.046), and Corsi Block-Tapping Task score (r = −0.136, *P* = 0.029). Patient age also directly correlated with AcroQoL total score (r = 0.209, *P* < 0.001), as well as with relative domains of physical (r = 0.168, *P* = 0.008), psychological (r = 0.217, *P* < 0.001), body image (r = 0.176, *P* = 0.005), and social relationship quality (r = 0.242, *P* < 0.001). Disease duration directly correlated with PSQI sleep (r = 0.172, *P* = 0.008) and total FSFI score (r = 0.205, *P* = 0.043). GH at diagnosis correlated directly with BUT-A body image score (r = 0.152, *P* = 0.025), and inversely with Corsi Block-Tapping Task score (r = −0.135, *P* = 0.038), and FAS score (r = −0.176, *P* = 0.014). IGF1 at evaluation correlated inversely with STAI Y 1 score (r = −0.122, *P* = 0.049). Similarly, IGF1 × ULN inversely correlated with STAI Y 1 score (r = −0.150, *P* = 0.016).

At the regression analysis, patient age was the best predictor of BDI somatic-affective domain score (t = −2.471, *P* = 0.014), STAI Y 1 score (t = 2.432, *P* = 0.016), BUT-A (t = −4.205, *P* < 0.001) total score and weight phobia (t = −4.934, *P* < 0.001), body image (t = −4.784, *P* < 0.001), avoidance (t = -2.982, *P* = 0.003), compulsive self-monitoring (t = −4.617, *P* < 0.001), and depersonalization (t = −3.728, *P* < 0.001) domain scores, BUT-B (t = −3.325, *P* < 0.001) and PSQI total scores t = −2.145, *P* = 0.033), and Corsi Block-Tapping Task score (t = −2.443, *P* = 0.016). GH at diagnosis was the best predictor of Berlin Questionnaire score (t = 2.794, *P* = 0.006) and Epworth Sleepiness Scale score (t = 4.028, *P* < 0.001). IGF1 at evaluation was the best predictor of S.T.A.Y 2 score (t = −2.714, *P* = 0.007).

As far as additional correlation studies among the different test scores are concerned, BDI-II total score correlated with S.T.A.Y 1 (r = 0.558, *P* < 0.001) and S.T.A.Y 2 (r = 0.520, *P* < 0.001) scores, PSQI score (r = 0.397, *P* < 0.001), BUT-A global score (r = 0.579, *P* < 0.001) and weight phobia (r = 0.526, *P* < 0.001), body image (r = 0.540, *P* < 0.001), avoidance (r = 0.547, *P* < 0.001), compulsive self-monitoring (r = 0.466, *P* < 0.001), and depersonalization (r = 0.531, *P* < 0.001) domain scores, BUT-B total score (r = 0.289, *P* < 0.001), as well as with AcroQoL total score (r = −0.661, *P* < 0.001), physical (r = −0.655, *P* < 0.001), psychological (r = −0.652, *P* < 0.001), body image (r = −0.602, *P* < 0.001), and social relationship quality (r = −0.565, *P* < 0.001) domain scores. BDI-II total score also correlated with Corsi Block-Tapping Task score (r = −0.137, *P* = 0.028) and Digit Span forward (r = −0.124, *P* = 0.046) score.

STAI Y 1 score correlated with PSQI (r = 0.296, *P* < 0.001), BUT-A total score (r = 0.483, *P* < 0.001) and with weight phobia (r = 0.434, *P* < 0.001), body image (r = 0.448, *P* < 0.001), avoidance (r = 0.451, *P* < 0.001), compulsive self-monitoring (r = 0.396, *P* < 0.001) and depersonalization (r = 0.441, *P* < 0.001) domain scores, BUT-B total score (r = 0.250, *P* < 0.001), as well as with AcroQoL total score (r = −0.553, *P* < 0.001), physical (r = −0.527, *P* < 0.001), psychological (r = −0.556, *P* < 0.001), body image (r = −-0.545, *P* < 0.001), and social relationship quality (r = −0.443, *P* < 0.001) domain scores. STAI Y 1 score also correlated with TMT-BA (r = -0.174, *P* = 0.006) and FAS (r = -0.134, *P* = 0.039) scores. STAI Y 2 score correlated with PSQI (r = 0.271, *P* < 0.001), BUT-A total score (r = 0.291, *P* < 0.001) and with weight phobia (r = 0.247, *P* < 0.001), body image (r = 0.291, *P* < 0.001), avoidance (r = 0.264, *P* < 0.001), compulsive self-monitoring (r = 0.243, *P* < 0.001) and depersonalization (r = 0.227, *P* < 0.001) domain scores, BUT-B total score (r = 0.20, *P* < 0.001), as well as with AcroQoL total score (r = −0.448, *P* < 0.001), physical (r = -0.443, *P* < 0.001), psychological (r = −0.439, *P* < 0.001), body image (r = −0.431, *P* < 0.001), and social relationship quality (r = −0.376, *P* < 0.001) domain scores.

PSQI score correlated with BUT-A total score (r = 0.4183, *P* = 0.003) and with weight phobia (r = 0.4123, *P* = 0.049), body image (r = 0.221, *P* < 0.001), avoidance (r = 0.261, *P* < 0.001) and depersonalization (r = 0.126, *P* < 0.043) domain scores, BUT-B total score (r = 0.199, *P* = 0.001), as well as with AcroQoL total score (r = −0.425, *P* < 0.001), physical (r = −0.441, *P* < 0.001), psychological (r = −0.356, *P* < 0.001), body image (r = −0.327, *P* < 0.001), and social relationship quality (r = −0.267, *P* < 0.001) domain scores. PSQI score also correlated with the Corsi Block-Tapping Task score (r = −0.123, *P* = 0.049) and Digit Span forward (r = −0.253, *P* < 0.001) score.

BUT-A total score correlated with AcroQoL total score (r = −0.494, *P* < 0.001), physical (r = −0.408, *P* < 0.001), psychological (r = −0.550, *P* < 0.001), body image (r = −0.497, *P* < 0.001), and social relationship quality (r = −0.495, *P* < 0.001) domain scores.

IIEF total score correlated with Berlin Questionnaire score (r = −0.224, *P* = 0.024, Epworth Sleepiness Scale score (r = −0.3, *P* = 0.002), and PSQI score (r = −0.357, *P* < 0.001), as well as with TMT-A score (r = −0.222, *P* = 0.025), TMT-B score (r = −0.204, *P* = 0.041), and FAS score (r = 0.214, *P* = 0.031). Similarly, FSFI total score correlated with TMT-A score (r = −0.474, *P* < 0.001), TMT-B score (r = −0.342, *P* < 0.001), and FAS score (r = 0.267, *P* = 0.004).

At regression analysis, BDI-II total score was the best predictor of AcroQoL physical domain score (t = −3.819, *P* < 0.001); PSQI was the best predictor of Digit Span forward score (t = −2.324, *P* = 0.021). Both BUT-A total score (t = −2.304, *P* = 0.022) and AcroQoL body image domain score (t = −2.494, *P* = 0.013) predicted the Corsi Block-Tapping Task score.

## Discussion

The current study demonstrated that psychological factors play a crucial role in the clinical course and management of acromegaly, impacting even in patients in long-term medical treatment, especially in absence of a clear disease control. Approximately 30% of acromegalic patients complained of a depressive condition resulting severe in 17% of cases and associated with a component of severe somatic-affective impairment in 15% and a component of severe cognitive decline in 17% of cases. Moreover, approximately 80% of patients were found to face an anxious mood, which was even worsened by a discomfort in body image, mainly registered in young women. As for the natural course of acromegaly, sleep disorders were found to occur in almost 60% of patients, together with daily sleepiness in approximately 20% of cases. Interestingly, a great majority of patients reported sexual dysfunction, with an erectile dysfunction registered in 60% of men, displaying a severe impairment in 25%, and a sexual dysfunction registered in 77% of women. Conversely, cognitive functions, including working memory, attention, and verbal fluency, were compromised in a minority of patients, accounting for approximately 10–15% of cases. These findings suggested that in addition to the somatic burden, acromegaly patients suffer from psychosocial and personality charges, contributing to impair the overall QoL.

Body and mind are strictly linked in acromegaly (7, 8, 9, 10), as in the majority of organic diseases, especially diseases with chronic and debilitating behaviour, such as acromegalic disease (11). Acromegaly patients gradually and inexorably experience body changes and loss of control of their body, which may become greatly different from the past life before disease occurrence, as a direct consequence of disease evolution *per se* or of the chronicity intrinsic in the acromegalic disease, thus resulting in remarkable psychological distress and increased psychological morbidity (7, 8, 9, 10). Noteworthy, the acromegalic disease is often misdiagnosed or diagnosed after a long delay (1), when long-term exposure to GH and IGF1 prompts the occurrence of permanent and life-threatening metabolic, cardiovascular, respiratory, skeletal, and neoplastic complications (5), known to exert a negative impact on a patient’s survival and life expectancy (43).

In this light, the psychological burden deriving from a chronic debilitating disease such as acromegaly contributes to a reduced QoL, and in addition acromegaly negatively impacts global psychological well-being, similarly to different systemic diseases. Consequently, acromegalic patients may experience depression in a non-neglectable proportion of cases, and psychological discomfort, characterized by anxiety, associated with low self-esteem, body image distortion, and social withdrawal, can be commonly registered (26, 27, 28). In the current study, depressive symptoms have been reported by approximately 30% of acromegalic patients, with more than half of them experiencing severe symptoms. Current findings are in line with a recent multicentric study, reporting that acromegalic patients suffered from probable depression in 28% of cases (44). In the current study, acromegaly was seen to exert a similar negative impact on both somatic-affective and cognitive domains of depression. Indeed, approximately one-third of patients displayed somatic-affective mood lowering and cognitive impairment, categorized as severe in half of the cases. Noteworthy, in the current experience, severe depression was found to occur predominantly in patients younger than 45 years or in those with a shorter disease duration, suggesting that in those individuals suddenly facing the diagnosis of a rare, chronic debilitating disease psychological bearing may be very pronounced. The inverse correlation between the patient’s age and the totality of depression scores strengthened the hypothesis that in younger patients the diagnosis of acromegaly may result in a direct negative impact on well-being perception and psychological health. Moreover, impressively, state and trait anxiety was reported by approximately 80% of acromegalic patients, but importantly from 40 to 50% of patients reported a clinically relevant anxiety with moderate or severe symptoms. The impact of patient age and disease duration on anxiety symptoms was found to parallel that of depression, as prevalence and severity of anxiety were higher in patients younger than 45 years or in those with a shorter disease duration. As for depression, the significant inverse correlation between the patient’s age and the totality of anxiety scores strengthened the hypothesis that in younger patients the diagnosis of acromegaly may result in a direct negative impact on anxiety, particularly moodiness and feeling scared about the future, therefore further supporting the idea that acromegaly may result in remarkable psychological distress. Conversely, gender, disease control, or concomitant comorbidities were found to exert no direct relevant impact on depression and anxiety prevalence and severity. Noteworthy, IGF1 levels were found inversely correlated with state anxiety; this finding, suggesting a state anxiety in patients with a greater disease control, need to be confirmed and better interpreted. Altogether, the current findings suggested that a recent diagnosis of acromegaly, mainly in young adults, by increasing awareness of a rare chronic debilitating disease, may result in unpleasant feelings or emotions affecting global well-being and severely limiting individual comfort in most patients.

Interestingly, depression and anxiety were found to persist in patients with long disease duration, or despite full biochemical control. A persistent negative illness perception, which might *per se* explain the depressive and anxious mood, has been reported also in patients with long-term remission of acromegaly (45). Such patients have been found to perceive a dramatic modification in body image during active disease, not completely recovered after long-term remission (45). The main determinants of body image discomfort have been reported to be female gender and young age at diagnosis (46). Consistent with these findings, in the current study, severe discomfort in body image was seen in approximately 20% of patients. Interestingly, a significantly worse perception of body image was recorded predominantly in female patients, younger than 45 years or with a disease duration less than 2 years, and patient age was found to be the best predictor of all BUT-A and BUT-B scores, thus confirming the significant influence of patient gender and age on body image concerns. Similar findings were seen in the totality of BUT-A domains, as weight phobia, body image, avoidance, compulsive self-monitoring, and depersonalization were worse in young female patients, with patient age being inversely correlated with the totality of BUT-A subscales and PSDI score. The significant correlation seen between BUT-A and AcroQoL total and subdomain scores suggested that the altered perception of body image may significantly influence the QoL. To a similar extent, BUT-B score directly correlated with BDI-II, STAI Y 1 and 2, and with PSQI, thus suggesting a strong link between impaired body image, depression, anxiety, and sleep disorders. Noteworthy, GH levels at diagnosis directly correlated with body image domain score, suggesting that disease activity, at least the disease activity at the time of disease discovery, might negatively influence body perception. Altogether, current findings led to the conclusion that acromegaly may directly and rapidly promote abnormal body image attitudes by inducing physical disfigurement and altering bodily self–perception, likely contributing to the development and persistence of anxiety and depression, and severely affecting QoL.

In the current study, depressive symptoms have been found associated with sleep impairment in patients with acromegaly. A strong link between depression and sleep has been extensively investigated (47). Most patients with depression, mainly women, display sleep disorders, including insomnia and hypersomnia, with a major impact on QoL and an increased risk for suicide (47). Sleep impairment may not recover with treatments for depression, with a greater risk of relapse and recurrence of sleep disorders (47). In turn, sleep impairment can exacerbate depression, and poor sleep status may even enhance depression, inducing a vicious circle between depression and sleep (48). Dopaminergic system, reportedly known to directly modulate GH synthesis and release at the pituitary level (49), has been proposed as one of the neurotransmitter systems implicated in the development and interplay of sleep disorders and depression (48), and dopaminergic agents are currently used in clinical practice to treat daytime sleepiness and sleep disorders (48). In the current study, sleep quality resulted severely impaired since around 60% of the patients were at high risk to develop OSAS and/or experiencing sleep disorders, and approximately 20% reported daily sleepiness. In fact, GH and IGF1 excess reportedly play a key role in OSAS pathogenesis in acromegaly, as it exerts direct effects on upper airway narrowing leading to mandible, maxilla, and ioid bones overgrowth, pharyngeal soft tissue swelling, and macroglossia (5), and indirect effects on cerebral breathing centres by altering the brain’s respiratory control centres (50). The results of the current study are in concordance with previous reports, demonstrating OSAS to occur in up to 78% of acromegaly patients (51, 52), with no significant impact of IGF1 levels, as expression of disease activity, on the prevalence and the severity of OSAS according to a recent meta-analysis, probably as a consequence of a partial and not complete resersibility of upper airways changes, induced by hormome excess, after hormone normalization (53). In line with these findings, in the current series, GH levels at diagnosis, but not IGF1 levels at the time of the evaluation, as expression of disease activity, were found to significantly influence OSAS and daily sleepiness, and GH at diagnosis emerged as the best predictor of the Berlin Questionnaire and Epworth Sleepiness Scale scores. Interestingly, depression scores were found to be directly correlated with PSQI scores, supporting a strong connection between depression and sleep disorders. Surprisingly, patient age inversely correlated with the prevalence and was the best predictor of the severity of sleep disorders; this finding, suggesting the occurrence of more and worse sleep disorders in young than in elderly patients, need to be confirmed and better interpreted. A relationship has been documented between either the severity or the frequency of apnoeas and male gender (5), as well as ageing and disease duration appear to be positively associated with the rate of apnoeic episodes (53, 54). In the current series, disease duration directly correlated with the prevalence of sleep disorders. Moreover, most patients with disease duration between 2 and 5 years were male, and this could *per se* explain the higher OSAS risk and sleep disorders seen in this group of patients. This finding might also contribute to explaining the greater impact on OSAS risk and sleep disorders in patients with disease duration between 2 and 5 years. In turn, a lower sleep quality was associated with a lower general well-being of patients, probably influencing the general quality of their life, the quality of their working activities, and the quality of their cognitive functions. This finding is also corroborated by the significant correlation seen between sleep disorders severity, as expressed by PSQI score, and AcroQoL total and physical, psychological, body image, and social relationship domains scores. Altogether, these results confirmed that in acromegaly sleep quality is poor and persistently compromised despite disease control, mainly in male patients with a recent diagnosis of acromegaly, in whom sleep disorders exert a negative impact on general well-being and QoL.

Sexual dysfunction was also documented in acromegalic patients in the current study, impacting on the QoL. Results from real-world studies have demonstrated that QoL is poorer in patients with sexual dysfunction and in their partners as compared to patients without sexual dysfunction due to relationship difficulties and decreased relationship satisfaction (55). In the current study, sexual dysfunction appeared to be slightly less compromised in men as compared to women. Particularly, men displayed erectile dysfunction in approximately 60% of cases, and a severe erectile dysfunction in 25% of cases; the data on general satisfaction indicated that men were partially satisfied, with 30% of patients unsatisfied with their own sexual experience. The impact of disease duration and patient age was clinically relevant to sexual function. In fact, approximately 56% of patients with disease duration less than 2 years displayed erectile dysfunction with impairment of general satisfaction. These findings might suggest that the diagnosis of a chronic debilitating disease, such as acromegaly, might severely affect sexual general satisfaction and erectile function immediately after the discovery of the disease. In the current study, disease control appeared to exert only a minor beneficial impact on the general quality of sexual function. The main determinant of sexual function in acromegalic men was age. As expected, older patients had worse scores in the totality of subdomains, raising the question of whether these results are induced directly by acromegaly *per se* or by the physiologic age-related decline in sexual function. Interestingly, GH levels at diagnosis, and IGF1 levels at evaluation, as expression slightly of disease control status, have been found not to significantly impact sexual function. A strong link between GH and IGF1 excess and sexual dysfunction in men has been documented (21, 22, 56, 57, 58, 59). The co-existence of hypogonadotropic hypogonadism due to tumour mass effect or treatments for acromegaly, such as neurosurgery and/or radiotherapy, and high levels of GH and IGF1 has been reported to alter the regular pulsatility of the gonadotropin secretion at the hypothalamic–pituitary level (57), thus contributing to the pathogenesis of erectile dysfunction as the expression of worse sexual health (21, 57, 58). Concomitant cardiovascular, such as arterial hypertension and endothelial dysfunction, respiratory, mainly OSAS, and metabolic complications, including abnormalities in glucose and lipid profile leading to insulin resistance, diabetes mellitus, and dyslipidaemia, may further compromise erectile function and induce a decrease of desire and arousability in acromegaly (21, 57). On the other hand, testosterone decline is known to occur physiologically in ageing men leading to the so-called late-onset or functional hypogonadism (60, 61, 62, 63, 64, 65, 66), and to act either as a cause or consequence of some chronic comorbidities, such as metabolic syndrome, diabetes mellitus, hypertension, and cardiovascular disease, commonly seen also in patients with acromegaly (60, 65, 67). In the current study, elderly patients reported lower IIEF total and sudomain scores, and the prevalence of erectile dysfunction was higher in older patients as compared to younger patients. The pathogenetic association of erectile dysfunction and patient age was also confirmed by the significant correlation of IIEF scores with a reduced attentive process, as expressed by all TMT scores, which was more prevalent in older patients. Additionally, IIEF scores were significantly correlated with risk of OSAS, daily sleepiness, and sleep disorders, as expressed by the Berlin Questionnaire, Epworth Sleepiness Scale and PSQI total scores, suggesting an association between sleep and sexual impairment in acromegaly. These findings are consistent with previous reports, documenting a significant association between OSAS and sexual dysfunction in male (68, 69, 70, 71, 72, 73, 74, 75, 76, 77, 78, 79, 80, 81, 82, 83, 84, 85, 86, 87) but not in female (88, 89, 90, 91, 92) patients. Similarly, the prevalence of erectile dysfunction was found significantly higher in patients with as compared to those without concomitant cardiovascular disease. Noteworthy, the IIEF questionnaire, as it is in the current form, can investigate men sexuality only in the last month, thus limiting the possibility to provide a fine and accurate definition of sexual function. In the current patient cohort, a minority of men (*n* = 10) had rare or absent sexual activity in the last month. Interestingly, only 3/10 patients were older than 64 years and declared that the absence of sexual activity was due to the recent widowhood state, whereas 7/10 patients were aged 45–64 years and declared that the absence of sexual activity was only due to the temporary condition of single status. Nevertheless, they all reported good or very good confidence to get an erection in previous relationships. The absence of sexual activity in these patients clearly contributed to worsening IIEF total and subdomain scores. However, based on the reported previous experience, it is likely that these men did not have a clinically relevant erectile dysfunction, and in these subjects, the investigation of additional items, such as presence of nocturnal erections or sexual activity over a longer timeframe than the month used in the IIEF questionnaire, could better refine estimates of erectile function. However, even taking into consideration the limitations of the IIEF questionnaire, the results of the current study suggested that in acromegalic men pathophysiology of erectile dysfunction appears to anticipate that of the general population, and older age and concomitant respiratory and cardiovascular diseases exert a negative impact on sexual health and promote a progressive decline in erectile function, desire, and arousability. Nevertheless, considering that the mean age of male patients in the current cohort was 54 years and that approximately one-quarter of them were >64 years, a bias directly related to the demographic characteristics of the patients cannot be excluded.

The findings on sexual function were even more striking in women, who displayed sexual dysfunction in approximately 80% of cases. The impact of disease duration and patient age was also clinically relevant to women sexual function. Women with a lower disease duration had higher desire scores as compared to those with longer disease duration, whereas FSFI total and arousal, lubrification, orgasm, satisfaction, and pain domain scores were similar among different disease duration groups. In turn, approximately 86% of patients with a disease duration more than 10 years displayed a sexual function impairment and had worsened functioning in the totality of FSFI subdomains. As expected, older patients had worse scores, and sexual dysfunction accounted for approximately 96% of women older than 64 years, raising the question of whether these results are induced directly by acromegaly *per se* or by the physiologic age-related decline in sexual function starting with menopause. Noteworthy, in patients with full disease control, desire scores were significantly higher than those of women with partial or absent disease control, whereas disease status did not significantly impact FSFI total and arousal, lubrification, orgasm, satisfaction, and pain scores. The link between GH and IGF1 excess and sexual dysfunction in women has been less extensively elucidated as compared to men. Paralleling the pathogenesis of sexual dysfunction in men, in acromegalic women the coexistence of an impaired gonadal function ascribable to hypogonadotropic hypogonadism due to tumour mass effect or treatments for acromegaly, such as neurosurgery and/or radiotherapy, together with additional common features of acromegaly in women such as hyperprolactinemia and/or polycystic ovary syndrome, may alter the regular pulsatility of the gonadotropin secretion at the hypothalamic–pituitary level (21). However, despite common pathophysiology, sexual health in acromegalic women has been less investigated compared to men, and available data are still very scant. Concomitant cardiovascular and metabolic complications may further disrupt sexual function in women and induce a decrease in arousability, orgasm, and satisfaction in acromegaly (21). On the other hand, the oestrogens fall spontaneously occurring with the onset of menopause leading to a state of physiologic hypogonadism, which is known to promote a cardiometabolic transition leading to obesity, metabolic syndrome, diabetes mellitus, arterial hypertension, and cardiovascular disease, commonly seen also in women with acromegaly (93, 94, 95). In the current study, older patients with longer disease duration reported lower FSFI total and all domain scores, and the prevalence of sexual dysfunction was higher in patients with longer disease duration and/or older age. Current findings are in line with previous studies on the general population, reporting an overall detrimental effect of menopause onset on women’s sexuality. Indeed, low sexual desire, poor lubrication, dyspareunia, and genitourinary syndrome have been reported to occur in up to 55% of menopausal women (96, 97). However, the considerably high prevalence of sexual dysfunction seen in the women of the current cohort may be ascribed to several additional factors, commonly found in acromegalic patients and potentially exacerbated by the menopausal transition. Together with the physiologic decline in sex steroids, cardiometabolic comorbidities and psychological relational changes related to ageing also play a key pathogenetic role (96). Consistent with these findings, the result of the present study demonstrated that the prevalence of sexual dysfunction was higher in women with concomitant cardiovascular disease as compared to those without this comorbidity, confirming the strong link between cardiometabolic and sexual health. To note, besides impaired sexual function, in the current experience, most elderly women also complained of mood disorders and body image distortion. The likelihood of depressed mood has been reported to be up to three times greater in the menopause transition compared with the premenopausal stage and to be influenced by psychosocial and lifestyle factors beyond hormonal changes (98). In the general population, the association between sexual dysfunction and mood disorders appears to be bidirectional (99). In addition to mood disorders, cognitive changes have been reported to occur frequently before, during, and after menopausal transition (100). Indeed, oestrogens are known to directly modulate brain function by regulating cell metabolism and increasing cerebral blood flow and dendritic outgrowth (100). In line with this evidence, in the current study, FSFI scores significantly correlated with cognitive decline, as expressed by a reduced attentive process at TMT testings. Altogether, these findings suggest that similarly to men, in acromegalic women, the pathophysiology of sexual dysfunction appears to anticipate that of the general population, and older age and concomitant cardiovascular disease exert a negative impact on sexual health. However, acromegalic women may experience a stronger negative influence of body image distortion and mood disorders on sexual health than men, in whom an individual’s body perception appears to exert a minimal pathogenetic role.

Interestingly, in both sexes, sexual dysfunction appeared to be associated with cognitive impairment. Indeed, the main cognitive activity was found slightly worsened in acromegaly patients. Particularly, visuospatial and verbal working memory, attentive process, and verbal fluency, troubles were found to occur in approximately 10–15% of patients in the current cohort. This might have a relevant influence on the general well-being of acromegaly patients, mainly concerning their self-efficacy and their self-esteem, with a likely effect on the emotional and personal dimensions (35, 36, 37, 38, 39, 40, 41, 42). Disease duration and patient age were found to be the main determinants of cognitive impairment in the current study. Indeed, the prevalence of visuospatial and verbal working memory, and attentive process, troubles was slightly higher in patients with longer disease duration and/or older age, confirming that also in acromegaly, together with disease duration, ageing is a main determinant of cognitive decline. Interestingly, the prevalence of verbal fluency troubles was higher in young adults aged < 45 years, raising the question of whether verbal fluency testing might help endocrinologists in the early detection of cognitive impairment in acromegalic patients. In fact, an association between cognitive decline and acromegaly can be postulated. Given the wide expression of GH and IGF1 receptors in different regions of the central nervous system involved in cognitive processes, such as the thalamus, hippocampus, and peri-hippocampal region (101), GH and IGF1 are known to act as modulators of cognitive functions (102), including processes of learning and memory, to prevent cell death during oxidative stress and ischemia and to stimulate neurogenesis at hippocampal level (103, 104). Based on this evidence, the disruption of GH/IGF1 axis occurring in acromegaly might represent *per se* a pathophysiologic mechanism responsible for cognitive impairment in acromegaly patients. However, a similar pathogenetic role for OSAS cannot be excluded. In fact, a strong association between OSAS and cognitive dysfunction has been extensively demonstrated, mainly involving executive function, attention, verbal/visual long-term memory, visuospatial and constructional ability, and information processing (105). Altogether, these findings suggested that in acromegaly cognitive decline is age-related but clinically trivial. In this light, extensive screening and monitoring of cognitive functions may be not required in all acromegalic patients, whereas a cognitive evaluation appears to be beneficial in older patients to detect specific cognitive deterioration.

In the current series, AcroQoL’s mean score was as high as 63.5, suggesting that patients in the present study, including patients treated and/or controlled for the disease, generally defined a non serious impairment of QoL in relation to the different specific domains explored by the questionnaire. Nevertheless, concomitant depression, anxiety, and body image distortion, as expressed by BDI-II, STAI Y 2, and BUT-A questionnaire scores, have been found to exert a significant impact on each specific domain of QoL. Previous studies have described an impaired QoL in patients with acromegaly. Noteworthy, controversial results have been reported, since the poor QoL seen at diagnosis of acromegaly has been demonstrated either to persist despite long-term remission regardless of the treatment used to achieve biochemical control (25, 106, 107, 108, 109) or to improve after GH and IGF1 normalization (110, 111, 112). Lower AcroQoL scores have been reported in patients with long disease duration (25, 108), multiple comorbidities (113), and in women (114). Partly in agreement with these findings, the results of the current study have demonstrated that in patients with partial disease control, AcroQoL scores, specifically those related to body image quality, were significantly lower as compared to patients with full or absent disease control, thus suggesting a limited role for the achievement of disease control on the full recovery of QoL in all specific domains. Additionally, in the current experience, concomitant arthralgia and osteoporosis further reduced physical quality scores. Disease duration and patient age were also found to impact the QoL in acromegaly. In the present study, the physical quality and the body image quality subscales were reduced, albeit not significantly, mainly in those patients with disease duration less than 2 years or of 2–5 years. Conversely, the social relationship quality seemed to improve proportionally to the disease duration and patient age. Therefore, patients with disease duration longer than 10 years, as well as patients older than 64 years, had a better social and relational attitude. To a similar extent, patient age directly correlated with AcroQoL total and physical, psychological, body image, and social relationship scores, with worse scores in the old as compared to young patients. Altogether, these findings suggest that multiple factors beyond hormonal levels and disease control, such as comorbidities, disease duration, and patient age, may negatively affect the QoL in acromegaly.

In conclusion, global psychological assessment, sleep quality, QoL, sexual function, and cognitive functions are severely compromised in acromegalic patients, despite medical therapy and independently of disease activity status. Disease duration, patient age, and gender play a crucial role in determining such a relevant impact on the psychological life of acromegaly patients, which appears to change across different age classes. Young patients and those recently diseased with acromegaly, who had to face the fear of progressive and uncontrolled body disfigurement peculiar to this chronic disease, generally experience depressive symptoms, anxiety, and body image distortion. In turn, older patients and those with long disease duration display a clinically relevant impairment in sexual function, which is prevalent in women, and in cognitive functions, particularly if some chronic comorbidities, mainly cardiometabolic and respiratory complications, exacerbate clinical features of acromegaly. The achievement of disease control cannot completely recover the psychological burden of acromegaly and is not always able to prevent poor QoL in patients with acromegaly. Future studies will help in clarifying the role and the burden of acromegaly *per se*, its comorbidities or other factors, such as ageing and menopause, in the pathogenesis and clinical management of psychological distress in acromegaly.

## Supplementary Material

Supplementary Material

## Declaration of interest

The authors declare that there is no conflict of interest that could be perceived as prejudicing the impartiality of the study reported.

## Funding

This study was partly supported by Ricerca Corrente funds from Fondazione IRCCS Ca’ Granda Ospedale Maggiore Policlinico, by grant NET 2018-12365454 from the Italian Ministry of Health http://dx.doi.org/10.13039/100009647, and by grant ID 41659099 from Pfizer http://dx.doi.org/10.13039/100004319.
