# Supplementary material for: Global psychological assessment with the evaluation of life and sleep quality and sexual and cognitive function in a large number of patients with acromegaly: a cross-sectional study
Source: Eur J Endocrinol. 2022 Sep 27;187(6):823–45. doi: 10.1530/EJE-22-0263 (PMC9782455; doi:10.1530/EJE-22-0263)
Supplement: Supplementary Material [file supplementary_material.pdf]

Supplemental Table 1: Results of the BDI-II questionnaire

**BDI-II**	**N=223**	**Score**
**Depression scale**		
Mean (SD)	65.9 (25.3)	Normal score <85
No depression	68.2%	score ≤ 85
Mild depression	9.9%	85-90
Moderate depression	4.5%	91-95
Severe depression	17.5%	score >95
**Somatic Affective scale**		
Mean (SD)	66.6 (24.3)	Normal score <85
No somatic-affective mood lowering	70%	score ≤ 85
Mild somatic-affective mood lowering	8.5%	85-90
Moderate somatic-affective mood lowering	6.3%	91-95
Severe somatic-affective mood lowering	15.2%	score >95
**Cognitive scale**		
Mean (SD)	71.7(21.2)	Normal score <85
No cognitive symptoms	60.5%	score ≤ 85
Mild cognitive symptoms	15.2%	85-90
Moderate cognitive symptoms	6.7%	91-95
Severe cognitive symptomatology	17.5%	score >95

**BDI-II by age range**	**<45 years (N=42)**	**45-64 years (N=125)**	**>64 years (N=56)**	**P-value**
**Depression scale**				

BDI-II by age range	<45 years (N=42)	45-64 years (N=125)	>64 years (N=56)	P-value
**Depression scale**				
Mean (SD)	74.5 (21.2)	64.3 (25.8)	63.0 (26.1)	0.047
Mild depression	7.1%	9.6%	12.5%	
Moderate depression	9.5%	4.0%	1.8%	0.37
Severe depression	23.8%	17.6%	12.5%	
**Somatic Affective scale**				
Mean (SD)	75.2 (19.8)	65.9 (24.5)	61.7 (25.6)	0.021
Mild somatic-affective mood lowering	9.5%	8.0%	8.9%	
Moderate somatic-affective mood lowering	7.1%	7.2%	3.6%	0.81
Severe somatic-affective mood lowering	21.4%	14.4%	12.5%	
**Cognitive scale**				
Mean (SD)	77.0 (19.9)	69.6 (21.5)	72.4 (21.2)	0.14
Mild cognitive symptoms	11.9%	16.0%	16.1%	
Moderate cognitive symptoms	11.9%	4.9%	7.1%	0.72
Severe cognitive symptoms	21.4%	16.0%	17.9%	

BDI-II by gender	Men (N=94)	Women (N=129)	P-value
**Depression scale**			
Mean (SD)	67.1 (26.2)	65.1(24.7)	0.57
Mild depression	11.7%	8.5%	
Moderate depression	6.4%	3.1%	0.42
Severe depression	19.1%	16.3%	
**Somatic Affective scale**			
Mean (SD)	65.8 (25.6)	65.0 (23.2)	0.26
Mild somatic-affective mood lowering	10.6%	7%	0.4
Moderate somatic-affective mood lowering	7.4%	5.4%	
Severe somatic-affective mood lowering	18.1%	13.2%	
**Cognitive scale**			

BDI-II by gender	Men (N=94)	Women (N=129)	P-value
Mean (SD)	73.5(19.5)	70.4 (22.4)	0.27
Mild cognitive symptoms	20.2%	11.6%	0.098
Moderate cognitive symptoms	9.6%	4.6%	
Severe cognitive symptoms	13.8%	20.2%	

BDI-II by disease duration	<2 year (N=25)	2-5 year (N=55)	5-10 year (N=59)	>10 year (N=52)	P-value
**Depression scale**					
Mean (SD)	72.0 (27.5)	68.9 (23.8)	59.7 (26.9)	66.7 (24.9)	0.13
Mild depression	16.0%	7.3%	6.8%	11.5%	0.39
Moderate depression	4%	9.1%	1.7%	5.8%	
Severe depression	28.0%	20.0%	13.6%	15.4%	
**Somatic Affective scale**					
Mean (SD)	72.5 (28.1)	69.5 (22.6)	61 (26.5)	66 (22.8)	0.16
Mild somatic-affective mood lowering	8.0%	5.4%	13.6%	5.8%	0.31
Moderate somatic-affective mood lowering	16.0%	7.3%	3.4%	5.8%	
Severe somatic-affective mood lowering	24.0%	18.2%	11.9%	13.5%	
**Cognitive scale**					
Mean (SD)	75.3 (21.6)	73.9 (20.5)	66.2 (22.9)	75.3 (20.2)	0.089
Mild cognitive symptoms	24.0%	16.4%	10.2%	19.2%	0.6
Moderate cognitive symptoms	12.0%	3.6%	5.1%	7.7%	
Severe cognitive symptoms	16.0%	21.8%	15.3%	19.2%	

BDI-II by disease control	Disease control (N=156)	No disease control (N=67)	P-value

BDI-II by disease control	Disease control (N=156)	No disease control (N=67)	P-value
**Depression scale**			
Mean (SD)	65.2 (25.2)	67.6 (25.6)	0.52
Mild depression	9%	11.9%	
Moderate depression	5.1%	3%	0.76
Severe depression	16.7%	19.4%	
**Somatic Affective scale**			
Mean (SD)	65.8 (24.1)	68.4 (24.8)	0.46
Mild somatic-affective mood lowering	8.3%	9%	0.037
Moderate somatic-affective mood lowering	3.2%	13.4%	
Severe somatic-affective mood lowering	16.0%	13.4%	
**Cognitive scale**			
Mean (SD)	71.2 (21.3)	72.7 (21)	0.63
Mild cognitive symptoms	15.4%	14.9%	
Moderate cognitive symptoms	7.0%	6%	0.96
Severe cognitive symptoms	16.7%	19.4%	

BDI-II by IGF-I categories	<=1 (N=156)	1.0-1.3 (N=32)	>1.3 (N=35)	P-value
**Depression scale**				
Mean (SD)	65.2 (25.2)	71.5 (25.5)	64.0 (25.5)	0.4
Mild depression	9%	12.5%	11.4%	
Moderate depression	5.1%	0.0%	5.7%	
Severe depression	16.7%	25.0%	14.3%	
**Somatic Affective scale**				
Mean (SD)	65.8 (24.1)	71.3 (24.3)	65.8 (25.2)	0.4
Mild somatic-affective mood lowering	8.3%	12.5%	5.7%	
Moderate somatic-affective mood lowering	3.2%	15.6%	11.4%	
Severe somatic-affective mood lowering	16.0%	12.5%	14.3%	
**Cognitive scale**				
Mean (SD)	71.2 (21.3)	76.8 (20.1)	69 (21.4)	0.4

**BDI-II by IGF-I categories**	**<=1 (N=156)**	**1.0-1.3 (N=32)**	**>1.3 (N=35)**	**P-value**
Mild cognitive symptoms	15.4%	15.6%	14.3%	
Moderate cognitive symptoms	7%	3.1%	8.6%	
Severe cognitive symptoms	16.7%	28.1%	11.4%	

Supplemental Table 2: Results of the the State-Trait Anxiety Inventory Form Y1 and Y2

**S.T.A.I. Y1 and Y2**	**N=223**	**Score**
**S.T.A.I. Y1**		
Mean (SD)	48.7 (11.8)	normal score ≤40
No state anxiety	17.7%	Score ≤40
Mild state anxiety	42.7%	Score 40-50
Moderate state anxiety	25%	Score 51-60
Severe state anxiety	14.6%	Score >60
**S.T.A.I. Y2**		
Mean (SD)	51.6 (12.8)	normal score ≤40
No trait anxiety	19.6%	Score ≤40
Mild trait anxiety	32.3%	Score 40-50
Moderate trait anxiety	26.5%	Score 51-60
Severe trait anxiety	21.5%	Score >60

**S.T.A.I. Y1 and Y2 by age class**	**<45 years (N=42)**	**45-64 years (N=125)**	**>64 years (N=56)**	**P-value**
**S.T.A.I. Y1**				
Mean (SD)	54.9 (11.9)	47.5 (11.4)	46.6 (11.4)	< 0.001
Absent/Mild state anxiety	21.4%	39.2%	41.1%	0.033
Moderate state anxiety	23.8%	32.0%	28.6%	
Severe state anxiety	54.8%	28.8%	30.4%	
**S.T.A.I. Y2**				
Mean (SD)	56.8 (12.3)	50.6 (12.1)	49.9 (13.9)	0.013
Absent/Mild trait anxiety	21.4%	36.0%	28.6%	0.095
Moderate trait anxiety	23.8%	32.8%	30.4%	
Severe trait anxiety	54.8%	31.2%	41.1%	

**S.T.A.I. Y1 and Y2 by gender**	**Male (N=94)**	**Female (N=129)**	**P-value**
**S.T.A.I. Y1**			

Mean (SD)	48.9 (14.1)	48.5 (9.9)	0.8
Absent/Mild state anxiety	31.9%	39.5%	
Moderate state anxiety	28.7%	30.2%	0.33
Severe state anxiety	39.4%	30.2%	
**S.T.A.I. Y2**			
Mean (SD)	52.5 (14.8)	50.9 (11.2)	0.37
Absent/Mild trait anxiety	31.9%	31.0%	
Moderate trait anxiety	28.7%	31.8%	0.88
Severe trait anxiety	39.4%	37.2%	

**S.T.A.I. Y1 and Y2 by disease duration**	**<2 years (N=25)**	**2-5 years (N=55)**	**5-10 years (N=59)**	**> 10 years (N=52)**	**P-value**
**S.T.A.I. Y1**					
Mean (SD)	48.8 (9.8)	50.6 (10.7)	46.9 (12.0)	46.9 (14.0)	0.31
Absent/Mild state anxiety	32.0%	34.5%	39.0%	46.2%	
Moderate state anxiety	32.0%	30.9%	35.6%	21.2%	0.63
Severe state anxiety	36.0%	34.5%	25.4%	32.7%	
**S.T.A.I. Y2**					
Mean (SD)	53.5 (11.9)	52.6 (10.2)	50.4 (13.1)	53.4 (12.7)	0.53
Absent/Mild trait anxiety	24.0%	25.5%	28.8%	34.6%	
Moderate trait anxiety	28.0%	38.2%	40.7%	23.1%	0.42
Severe trait anxiety	48.0%	36.4%	30.5%	42.3%	

**S.T.A.I. Y1 and Y2 by disease control**	**Disease control (N=156)**	**No disease control (N=67)**	**P-value**
**S.T.A.I. Y1**			
Mean (SD)	49.4 (11.3)	46.9 (12.9)	0.18

S.T.A.I. Y1 and Y2 by disease control	Disease control (N=156)	No disease control (N=67)	P-value
Absent/Mild state anxiety	34%	41.8%	
Moderate state anxiety	29.5%	29.9%	0.43
Severe state anxiety	36.5%	28.4%	
**S.T.A.I. Y2**			
Mean (SD)	51.3 (13.0)	52.3 (12.5)	0.62
Absent/Mild trait anxiety	29.5%	35.8%	0.12
Moderate trait anxiety	34.6%	20.9%	
Severe trait anxiety	35.9%	43.3%	

S.T.A.I. Y1 and Y2 by IGF-I categories	<=1 (N=156)	1.0-1.3 (N=32)	>1.3 (N=35)	P-value
**S.T.A.I. Y1**				
Mean (SD)	49.4 (11.3)	47.2 (12.2)	46.7 (13.7)	0.35
Absent/Mild state anxiety	34%	31.3%	51.4%	
Moderate state anxiety	29.5%	37.5%	22.9%	0.29
Severe state anxiety	36.5%	31.3%	25.7%	
**S.T.A.I. Y2**				
Mean (SD)	51.3 (13.0)	52.4 (11.4)	52.1 (13.5)	0.88
Absent/Mild trait anxiety	29.5%	28.1%	42.9%	
Moderate trait anxiety	34.6%	21.9%	20.0%	0.19
Severe trait anxiety	35.9%	50.0%	37.1%	

Supplemental Table 3: Results of the Body Uneasiness Test A and B

BUT A – BUT B	N=223	Score
**BUT A GSI (Global Severity Index)**		
Mean (SD)	0.8 (0.9)	normal value ≤1.2
No global concern	78.7%	≤1.2
Global concern	21.3%	>1.2
**Weight_Phobia**		
Mean (SD)	1.0 (1.1)	normal value <1
No Concern	67.4%	<1
Concern	32.6%	>1
**Body Image Concerns**		
Mean (SD)	1.0 (1.0)	normal value <1
No Concern	62.9%	<1
Concern	37.1%	>1
**Avoidance**		
Mean (SD)	0.5 (0.8)	normal value <1
No Concern	85.1%	<1
Concern	14.9%	>1
**Compulsive_Self_Monitoring**		
Mean (SD)	0.7 (0.8)	normal value <1
No Concern	71.9%	<1
Concern	28.1%	>1
**Depersonalization**		
Mean (SD)	0.4 (0.9)	normal value <1
No Concern	86%	<1
Concern	14%	>1
**BUT B Positive Symptom Distress Index (PSDI)**		
Mean (SD)	2.1 (1.2)	normal value <1
**BUT B 1 Mouth**		
Mean (SD)	0.9 (1.0)	normal value <1
**BUT B 2 Face**		
Mean (SD)	1.2 (0.9)	normal value <1
**BUT B 3 Things**		

Mean (SD)	0.8 (1.2)	normal value <1
**BUT B 4 Legs**		
Mean (SD)	1.0 (1.1)	normal value <1
**BUT B 5 Arms**		
Mean (SD)	0.5 (0.9)	normal value <1
**BUT B 6 Moustache**		
Mean (SD)	0.5 (0.9)	normal value <1
**BUT B 7 Skin**		
Mean (SD)	0.7 (1.2)	normal value <1
**BUT B 8 Blushing**		
Mean (SD)	0.6 (0.9)	normal value <1

**BUT A – BUT B by age class**	**<45 years (N=42)**	**45-64 years (N=125)**	**>64 years (N=56)**	**P-value**
**BUT A GSI (Global Severity Index)**				
Mean (SD)	1.4 (1.0)	0.8 (0.8)	0.4 (0.4)	< 0.001
No global concern	52.4%	81.5%	92.7%	< 0.001
Global concern	47.6%	18.5%	7.3%	
**Weight_Phobia (WP)**				
Mean (SD)	1.7 (1.3)	1.0 (1.1)	0.4 (0.6)	< 0.001
No Concern	38.1%	67.7%	89.1%	< 0.001
Concern	61.9%	32.3%	10.9%	
**Body Image Concerns (BIC)**				
Mean (SD)	1.8 (1.2)	1.0 (1.0)	0.6 (0.6)	< 0.001
No Concern	35.7%	63.7%	81.8%	< 0.001
Concern	64.3%	36.3%	18.2%	
**Avoidance**				
Mean (SD)	0.8 (0.9)	0.4 (0.7)	0.3 (0.5)	0.002
No Concern	76.2%	87.9%	85.5%	0.18
Concern	23.8%	12.1%	14.5%	
**Compulsive_Self_Monitoring**				
Mean (SD)	1.3 (1.0)	0.7 (0.8)	0.3 (0.4)	<0.001
No Concern	45.2%	72.6%	90.9%	< 0.001

Concern	54.8%	27.4%	9.1%	
**Depersonalization**				
Mean (SD)	0.9 (1.1)	0.4 (0.9)	0.1 (0.3)	<0.001
No Concern	66.7%	87.9%	96.4%	<0.001
Concern	33.3%	12.1%	3.6%	
**BUT B Positive Symptom Distress Index (PSDI)**				
Mean (SD)	2.6 (1.2)	2.1 (1.2)	1.9 (1.1)	0.006
**BUT B 1 Mouth**				
Mean (SD)	1.3 (1.3)	0.8 (1.0)	0.6 (0.6)	0.003
**BUT B 2 Face**				
Mean (SD)	1.0 (1.3)	0.63 (0.8)	0.33 (0.5)	< 0.001
**BUT B 3 Thighs**				
Mean (SD)	1.4 (1.5)	0.8 (1.1)	0.4 (0.7)	< 0.001
**BUT B 4 Legs**				
Mean (SD)	1.5 (1.2)	1.0 (1.1)	0.6 (0.7)	< 0.001
**BUT B 5 Arms**				
Mean (SD)	1.0 (1.2)	0.5 (0.9)	0.2 (0.4)	< 0.001
**BUT B 6 Moustache**				
Mean (SD)	0.8 (1.1)	0.4 (0.9)	0.3 (0.7)	0.013
**BUT B 7 Skin**				
Mean (SD)	1.1 (1.5)	0.8 (1.2)	0.5 (0.9)	0.046
**BUT B 8 Blushing**				
Mean (SD)	1.1 (1.1)	0.6 (0.9)	0.4 (0.6)	< 0.001

BUT A – BUT B by gender	Male (N=94)	Female (N=129)	P-value
**BUT A GSI (Global Severity Index)**			
Mean (SD)	0.6 (0.6)	0.9 (1.0)	0.004
No global concern	83.9%	75.0%	0.15
Global concern	16.1%	25.0%	
**Weight_Phobia**			
Mean (SD)	0.7 (0.8)	1.2 (1.2)	<0.001
No Concern	72.0%	64.1%	0.27

Concern	28.0%	35.9%	
**Body Image Concerns**			
Mean (SD)	0.8 (0.9)	1.2 (1.1)	0.006
No Concern	68.8%	58.6%	0.16
Concern	31.2%	41.4%	
**Avoidance**			
Mean (SD)	0.4 (0.6)	0.6 (0.8)	0.038
No Concern	87.1%	83.6%	0.6
Concern	12.9%	16.4%	
**Compulsive_Self_Monitoring**			
Mean (SD)	0.6 (0.7)	0.9 (0.9)	0.008
No Concern	76.3%	68.7%	0.28
Concern	23.7%	31.3%	
**Depersonalization**			
Mean (SD)	0.3 (0.6)	0.95 (1.0)	0.024
No Concern	90.3%	82.8%	0.16
Concern	9.7%	17.2%	
**BUT B Positive Symptom Distress Index (PSDI)**			
Mean (SD)	1.9 (1.2)	2.4 (1.1)	< 0.001
**BUT B 1 Mouth**			
Mean (SD)	0.7 (0.9)	1.0 (1.0)	0.059
**BUT B 2 Face**			
Mean (SD)	0.6 (0.9)	0.7 (0.9)	0.63
**BUT B 3 Thighs**			
Mean (SD)	0.4 (0.8)	1.1 (1.3)	< 0.001
**BUT B 4 Legs**			
Mean (SD)	0.6 (0.8)	1.3 (1.1)	< 0.001
**BUT B 5 Harms**			
Mean (SD)	0.3 (0.7)	0.7 (1.0)	0.002
**BUT B 6 Moustache**			
Mean (SD)	0.3 (0.7)	0.6 (1.0)	0.011
**BUT B 7 Skin**			
Mean (SD)	0.6 (1.1)	0.9 (1.2)	0.08

**BUT B 8 Blushing**			
Mean (SD)	0.4 (0.7)	0.8 (1.0)	< 0.001

BUT A – BUT B by disease duration	<2 year (N=25)	2-5 year (N=55)	5-10 year (N=59)	>10 year (N=52)	P-value
**BUT A GSI (Global Severity Index)**					
Mean (SD)	1.1 (1.0)	0.8 (0.8)	0.6 (0.7)	0.8 (1.0)	0.17
No global concern	64.0%	76.4%	87.7%	82.7%	0.079
Global concern	36.0%	23.6%	12.3%	17.3%	
**Weight_Phobia**					
Mean (SD)	1.4 (1.4)	1.0 (1.1)	0.8 (0.8)	0.6 (0.9)	0.095
No Concern	56.0%	63.6%	70.2%	75.0%	0.33
Concern	44.0%	36.4%	29.8%	25.0%	
**Body Image Concerns**					
Mean (SD)	1.3 (1.2)	1.1 (1.0)	0.9 (0.9)	1.0 (1.1)	0.35
No Concern	56.0%	54.5%	71.9%	65.4%	0.23
Concern	44.0%	45.5%	28.1%	34.6%	
**Avoidance**					
Mean (SD)	0.6 (0.8)	0.4 (0.7)	0.9 (0.6)	0.5 (0.9)	0.58
No Concern	80.0%	83.6%	87.7%	86.5%	0.8
Concern	20.0%	16.4%	12.3%	13.5%	
**Compulsive_Self_Monitoring**					
Mean (SD)	0.6 (0.9)	0.6 (0.9)	0.6 (0.9)	0.6 (0.9)	0.29
No Concern	52.0%	72.7%	82.5%	71.2%	0.043
Concern	48.0%	27.3%	17.5%	28.8%	
**Depersonalization**					
Mean (SD)	0.6 (1.0)	0.4 (0.7)	0.3 (0.8)	0.5 (1.1)	0.5
No Concern	76.0%	87.3%	91.2%	84.6%	0.31
Concern	24.0%	12.7%	8.8%	15.4%	
**BUT B Positive Symptom Distress Index (PSDI)**					
Mean (SD)	2.22 (1.38)	2.28 (1.19)	2.08 (1.24)	2.15 (1.29)	0.86
**BUT B 1 Mouth**					

Mean (SD)	0.7 (1.0)	0.7 (0.6)	1.0 (1.1)	1.0 (1.2)	0.32
**BUT B 2 Face**					
Mean (SD)	0.6 (0.9)	0.6 (0.7)	0.5 (0.9)	0.8 (1.0)	0.48
**BUT B 3 Things**					
Mean (SD)	0.7 (1.2)	0.7 (1.1)	0.8 (1.02)	0.9 (1.3)	0.88
**BUT B 4 Legs**					
Mean (SD)	1.1 (1.1)	0.9 (1.0)	0.9 (1.0)	1.0 (1.2)	0.81
**BUT B 5 Arms**					
Mean (SD)	0.7 (1.0)	0.5 (0.9)	0.5 (0.9)	0.5 (0.9)	0.67
**BUT B 6 Moustache**					
Mean (SD)	0.3 (0.7)	0.5 (0.8)	0.5 (1.2)	0.4 (0.8)	0.72
**BUT B 7 Skin**					
Mean (SD)	0.9 (1.3)	0.7 (1.2)	0.7 (1.01)	0.6 (1.1)	0.74
**BUT B 8 Blushing**					
Mean (SD)	0.6 (0.7)	0.5 (0.6)	0.7 (1.0)	0.7 (1.0)	0.59

**BUT A – BUT B by disease control**	**Disease control (N=156)**	**No disease control (N=67)**	**P-value**
**BUT A GSI (Global Severity Index)**			
Mean (SD)	0.8 (0.9)	0.8 (0.8)	0.73
No global concern	79.9%	76.1%	0.65
Global concern	20.1%	23.9%	
**Weight_Phobia**			
Mean (SD)	1.0(1.3)	1.0 (1.1)	0.58
No Concern	69.5%	62.7%	0.4
Concern	30.5%	37.3%	
**Body Image Concerns**			
Mean (SD)	1.0 (1.1)	1.0 (1.0)	0.68
No Concern	64.9%	58.2%	0.42
Concern	35.1%	41.8%	
**Avoidance**			
Mean (SD)	0.5 (1.0)	0.4 (0.6)	0.17

No Concern	83.8%	88.1%	0.54
Concern	16.2%	11.9%	
**Compulsive_Self_Monitoring**			
Mean (SD)	0.7 (0.8)	0.8 (0.9)	0.26
No Concern	73.4%	68.7%	0.58
Concern	26.6%	31.3%	
**Depersonalization**			
Mean (SD)	0.4 (0.9)	0.5 (0.8)	0.7
No Concern	87.0%	83.6%	0.64
Concern	13.0%	16.4%	
**BUT B Positive Symptom Distress Index (PSDI)**			
Mean (SD)	2.07 (1.2)	2.3(1.2)	0.28
**BUT B 1 Mouth**			
Mean (SD)	0.8 (1.0)	0.9 (1.0)	0.46
**BUT B 2 Face**			
Mean (SD)	0.6 (0.9)	0.7 (0.9)	0.46
**BUT B 3 Thighs**			
Mean (SD)	0.8(1.2)	0.9 (1.1)	0.35
**BUT B 4 Legs**			
Mean (SD)	0.9 (1.1)	1.1 (1.1)	0.39
**BUT B 5 Arms**			
Mean (SD)	0.6 (1.0)	0.6 (0.7)	0.41
**BUT B 6 Moustache**			
Mean (SD)	0.4 (0.9)	0.5 (0.78)	0.83
**BUT B 7 Skin**			
Mean (SD)	0.7 (1.2)	0.8 (1.2)	0.76
**BUT B 8 Blushing**			
Mean (SD)	0.6 (0.9)	0.68 (0.9)	0.21

BUT A – BUT B by IGF-I class	<=1 (N=156)	1.0-1.3 (N=32)	>1.3 (N=35)	P-value
**BUT A GSI (Global Severity Index)**				
Mean (SD)	0.8 (0.9)	0.7 (0.8)	0.9 (0.8)	0.8
No global concern	79.9%	81.2%	71.4%	0.51
Global concern	20.1%	18.8%	28.6%	
**Weight_Phobia**				
Mean (SD)	0.9 (1.1)	1.0 (1.1)	1.1 (1.1)	0.84
No Concern	69.5%	62.5%	62.9%	0.61
Concern	30.5%	37.5%	37.1%	
**Body Image Concerns**				
Mean (SD)	1.0 (1.1)	1.0 (0.9)	1.2 (1.1)	0.79
No Concern	64.9%	53.1%	62.9%	0.45
Concern	35.1%	46.9%	37.1%	
**Avoidance**				
Mean (SD)	0.5 (0.8)	0.4 (0.7)	0.4 (0.5)	0.45
No Concern	83.8%	90.6%	85.7%	0.61
Concern	16.2%	9.4%	14.3%	
**Compulsive_Self_Monitoring**				
Mean (SD)	0.7 (0.8)	0.8 (0.9)	0.9 (1.0)	0.48
No Concern	73.4%	71.9%	65.7%	0.66
Concern	26.6%	28.1%	34.3%	
**Depersonalization**				
Mean (SD)	0.4 (0.9)	0.4 (0.8)	0.5 (0.9)	0.84
No Concern	87.0%	90.6%	77.1%	0.23
Concern	13.0%	9.4%	22.9%	
**BUT B Positive Symptom Distress Index (PSDI)**				
Mean (SD)	2.1 (1.2)	2.3 (1.3)	2.2 (1.2)	0.52
**BUT B 1 Mouth**				
Mean (SD)	0.8 (1.0)	1.0 (1.1)	0.9 (1.0)	0.58
**BUT B 2 Face**				
Mean (SD)	0.6 (0.9)	0.8 (0.9)	0.6 (0.9)	0.48
**BUT B 3 Thighs**				

Mean (SD)	0.8 (1.2)	1.0 (1.1)	0.8 (1.1)	0.56
**BUT B 4 Legs**				
Mean (SD)	0.9 (1.1)	1.2 (1.1)	1.0 (1.0)	0.47
**BUT B 5 Arms**				
Mean (SD)	0.5 (0.9)	0.8 (0.9)	0.4 (0.5)	0.27
**BUT B 6 Moustache**				
Mean (SD)	0.4 (0.9)	0.6 (0.9)	0.3 (0.7)	0.53
**BUT B 7 Skin**				
Mean (SD)	0.7 (1.2)	0.9 (1.3)	0.7 (1.1)	0. 6
**BUT B 8 Blushing**				
Mean (SD)	0.6 (0.9)	0.9 (1.1)	0.6 (0.7)	0.25

Supplemental Table 4: Results of the Berlin questionnaire, Epworth Sleepiness Scale and Pittsburgh Sleep Quality Inventory

**SLEEP QUALITY**	**N=223**	**Scores**
**BERLIN QUESTIONNAIRE**		
Absent or Low risk to develop OSAS	40.8%	normal score ≤1
High Risk to develop OSAS	59.2%	>1
**Epworth Sleepiness Scale**		
Normal daily sleepiness	80.7%	normal score ≤10
Mild daily sleepiness	12.9%	11-12
Moderate daily sleepiness	6.4%	13-15
Severe daily sleepiness	0%	16-24
**Pittsburgh Sleep Quality Inventory (PSQI)**		
No sleep disorders	39.0%	normal score ≤5
Sleep disorders	61.0%	>5

**SLEEP QUALITY by age class**	**<45 years (N=42)**	**45-64 years (N=125)**	**>64 years (N=56)**	**P-value**
**BERLIN QUESTIONNAIRE**				
Absent or Low risk to develop OSAS	47.6%	39.2%	39.3%	0.61
High risk to develop OSAS	52.4%	60.8%	60.7%	
**Epworth Sleepiness Scale**				
Normal daily sleepiness	81.0%	80.0%	82.1%	0.94
Anormal daily sleepiness	19.0%	20.0%	17.9%	
**Pittsburgh Sleep Quality Inventory (PSQI)**				
No sleep disorders	45.2%	39.2%	33.9%	0.52
Sleep disorders	54.8%	60.8%	66.1%	

**SLEEP QUALITY by gender**	**Male (N=94)**	**Female (N=129)**	**P-value**
**BERLIN QUESTIONNAIRE**			
Absent or Low risk to develop OSAS	36.2%	44.2%	0.29
High risk to develop OSAS	63.8%	55.8%	

**Epworth Sleepiness Scale**			
Normal daily sleepiness	77.7%	82.9%	0.41
Anormal daily sleepiness	22.3%	17.1%	
**Pittsburgh Sleep Quality Inventory (PSQI)**			
No sleep disorders	42.6%	36.4%	0.43
Sleep disorders	57.4%	63.6%	

SLEEP QUALITY by disease duration	<2 year (N=25)	2-5 year (N=55)	5-10 year (N=59)	>10 year (N=52)	P-value
**BERLIN QUESTIONNAIRE**					
Absent or Low risk to develop OSAS	56.0%	32.7%	47.5%	34.6%	0.12
High risk to develop OSAS	44.0%	67.3%	52.5%	65.4%	
**Epworth Sleepiness Scale**					
Normal daily sleepiness	84.0%	72.7%	83.1%	80.8%	0.5
Abnormal daily sleepiness	16.0%	27.3%	16.9%	19.2%	
**Pittsburgh Sleep Quality Inventory**					
No sleep disorders	48.0%	36.4%	39.0%	42.3%	0.78
Sleep disorders	52.0%	63.6%	61.0%	57.7%	

SLEEP QUALITY by disease control	Disease control (N=156)	No disease control (N=67)	P-value
**BERLIN QUESTIONNAIRE**			
Absent or Low risk to develop OSAS	41.0%	40.3%	0.99
High riskisk to develop OSAS	59.0%	59.7%	
**Epworth Sleepiness Scale**			
Normal daily sleepiness	80.8%	80.6%	0.99
Anormal daily sleepiness	19.2%	19.4%	
**Pittsburgh Sleep Quality Inventory (PSQI)**			
No sleep disorders	36.5%	44.8%	0.31
Sleep disorders	63.5%	55.2%	

SLEEP QUALITY by IGF-I class	<=1 (N=156)	1.0-1.3 (N=32)	>1.3 (N=35)	P-value
**BERLIN QUESTIONNAIRE**				
Absent or Low risk to develop OSAS	41.0%	34.4%	45.7%	0.64
High risk to develop OSAS	59.0%	65.6%	54.3%	
**Epworth Sleepiness Scale**				
Normal daily sleepiness	80.8%	78.1%	82.9%	0.89
Anormal daily sleepiness	19.2%	21.9%	17.1%	
**Pittsburgh Sleep Quality Inventory (PSQI)**				
No sleep disorders	36.5%	40.6%	48.6%	0.41
Sleep disorders	63.5%	59.4%	51.5%	

Supplemental Table 5: Evaluation of sexual function in men with the International Index for Erectile Function (A) and women with theFemale Sexual Function Index (B)

A

MEN SEXUAL FUNCTION	N=94	Score
**IIEF total score**		
Mean (SD)	45.6 (23.8)	Score 5-75
**Orgasm**		
Mean (SD)	6.8 (4.5)	Score 0-10
**Desire**		
Mean (SD)	6.5 (3.5)	Score 2-10
**Intercourse satisfaction**		
Mean (SD)	7.5 (4.0)	Score 0-15
**General satisfaction**		
Mean (SD)	6.3 (3.0)	Score 2-10
Very satisfied	46.2%	10
Moderately Satisfied	23 %	8
Unsatisfied	4.4%	4
Very unsatisfied	26.4%	2
**Erectile Function**		Score 1-30
Mean (SD)	18.4 (11.1)	Normal score 26-30
No Erectile dysfunction	40.4%	
Mild Erectile dysfunction	22.3%	17-25
Moderate Erectile dysfunction	12.8%	11-16
Severe Erectile dysfunction	24.5%	6-10

MEN SEXUAL FUNCTION by age class	<45 years (N=18)	45-64 years (N=52)	>64 years (N=24)	P-value
**IIEF total score**				
Mean (SD)	52.1 (20.5)	50.5 (20.3)	25.6 (26.3)	< 0.001
**Erectile Function**				
Mean (SD)	21.6 (9.1)	20.5 (10.1)	9.5 (11.4)	< 0.001
**Orgasm**				
Mean (SD)	7.3 (4.0)	7.9 (3.9)	3.3 (4.7)	< 0.001

**Desire**				
Mean (SD)	7.6 (2.8)	7.2 (2.9)	3.5 (3.9)	< 0.001
**Intercourse satisfaction**				
Mean (SD)	8.4 (4.2)	8.0 (3.7)	5.1 (4.1)	0.012
**General satisfaction**				
Mean (SD)	7.2 (2.7)	6.8 (2.6)	4.0 (3.3)	< 0.001
**Erectile dysfunction**				
No erectile dysfunction	61.1%	48.1%	29.2%	
Mild Erectile dysfunction	11.1%	26.9%	8.3%	0.021
Moderate Erectile dysfunction	11.1%	15.4%	0%	
Severe Erectile dysfunction	16.7%	9.6%	62.5%	

MEN SEXUAL FUNCTION by disease duration	<2 year (N=18)	2-5 year (N=17)	5-10 year (N=29)	>10 year (N=30)	P-value
**IIEF total score**					
Mean (SD)	34.6 (27.4)	49.1 (21.4)	48.7 (22.9)	45.3 (25.5)	0.32
**Erectile Function**					
Mean (SD)	14.4 (12.5)	19.5 (10.4)	19.6 (11.6)	18.0 (11.3)	0.56
**Orgasm**					
Mean (SD)	5.00 (5.3)	7.8 (3.7)	7.1 (4.2)	6.9 (5.0)	0.35
**Desire**					
Mean (SD)	5.00 (3.8)	6.9 (3.26)	6.5 (2.8)	6.6 (4.4)	0.47
**Intercourse satisfaction**					
Mean (SD)	5.2 (4.2)	8.1 (3.4)	8.2 (4.1)	7.7 (3.9)	0.13
**General satisfaction**					
Mean (SD)	5.0 (2.8)	6.79 (3.0)	7.00 (2.8)	6.1 (3.2)	0.24
**Erectile dysfunction**					
No erectile dysfunction	44.4%	29.5%	48.3%	40%	0.64

Mild Erectile dysfunction	16.7%	29.4%	13.8%	23.3%
Moderate Erectile dysfunction	11.1%	23.5%	13.8%	6.7%
Severe Erectile dysfunction	27.8%	17.6%	24.1%	30%

**MEN SEXUAL FUNCTION by disease control**	**Disease control (N=70)**	**No disease control (N=24)**	**P-value**
**IIEF total score**			
Mean (SD)	46.2 (23.5)	43.4 (25.3)	0.65
**Erectile Function**			
Mean (SD)	18.9 (10.9)	16.7 (11.8)	0.45
**Orgasm**			
Mean (SD)	6.7 (4.5)	7.2 (4.2)	0.67
**Desire**			
Mean (SD)	6.4 (3.4)	6.8 (3.8)	0.71
**Intercourse satisfaction**			
Mean (SD)	7.7 (3.7)	6.8 (4.8)	0.41
**General satisfaction**			
Mean (SD)	6.4 (2.9)	6.0 (3.4)	0.65
**Erectile dysfunction**			
No erectile dysfunction	47.2%	12.5%	0.006
Mild Erectile dysfunction	21.4%	16.7%	
Moderate Erectile dysfunction	8.6%	16.7%	
Severe Erectile dysfunction	22.8%	54.1%	

MEN SEXUAL FUNCTION by IGF-I class	<=1 (N=70)	1.0-1.3 (N=12)	>1.3 (N=12)	P-value
**IIEF total score**				
Mean (SD)	46.2 (23.5)	46.8 (26.5)	38.9 (24.5)	0.68
**Erectile Function**				
Mean (SD)	18.9 (10.9)	18.3 (12.5)	14.4 (11.1)	0.53
**Orgasm**				
Mean (SD)	6.7 (4.5)	7.7 (4.5)	6.4 (3.9)	0.74
**Desire**				
Mean (SD)	6.4 (3.4)	7.7 (4.0)	5.6 (3.3)	0.36
**Intercourse satisfaction**				
Mean (SD)	7.7 (3.7)	6.7 (5.1)	6.8 (4.8)	0.63
**General satisfaction**				
Mean (SD)	6.4 (2.9)	6.2 (3.3)	5.7 (3.7)	0.81
**Erectile dysfunction**				
No erectile dysfunction	47.2%	25%	33.3%	
Mild Erectile dysfunction	21.4%	16.7%	16.7%	0.12
Moderate Erectile dysfunction	8.6%	8.3%	25%	
Severe Erectile dysfunction	22.8%	50%	25%	

B

WOMEN SEXUAL FUNCTION	N=129	Score
**FSFI total score**		
Mean (SD)	15.7 (11.3)	Normal score ≥ 26.55
**Desire**		
Mean (SD)	3.0 (1.6)	score 1.2-6
**Arousal**		
Mean (SD)	2.3 (2.0)	score 0-6
**Lubrication**		

Mean (SD)	2.6 (2.3)	score 0-6
**Orgasm**		
Mean (SD)	2.5 (2.3)	score 0-6
**Satisfaction**		
Mean (SD)	3.0 (1.8)	score 0.8-6
**Pain**		
Mean (SD)	2.7 (2.6)	score 0-6
Sexual dysfunction	77.5%	
No Sexual dysfunction	22.5%	

**WOMEN SEXUAL FUNCTION by age class**	**<45 years (N=24)**	**45-64 years (N=72)**	**>64 years (N=33)**	**P-value**
**FSFI total score**				
Mean (SD)	20.0 (11.6)	17.0 (10.7)	8.0 (8.5)	< 0.001
**Desire**				
Mean (SD)	3.4 (1.3)	3.1 (1.6)	2.3 (1.5)	0.034
**Arousal**				
Mean (SD)	2.9 (1.9)	2.6 (2.0)	1.0 (1.5)	< 0.001
**Lubrication**				
Mean (SD)	3.5 (2.3)	2.9 (2.3)	0.8 (1.2)	< 0.001
**Orgasm**				
Mean (SD)	3.5 (2.3)	2.7 (2.2)	0.8 (1.2)	< 0.001
**Satisfaction**				
Mean (SD)	3.8 (1.7)	3.0 (1.8)	2.1 (1.6)	0.004
**Pain**				
Mean (SD)	3.5 (2.4)	2.8 (2.5)	1.4 (2.4)	0.012
Sexual dysfunction	60.0%	77.4%	95.8%	0.011
No Sexual dysfunction	40.0%	22.6%	4.2%	

**WOMEN SEXUAL FUNCTION by disease duration**	**<2 year (N=28)**	**2-5 year (N=31)**	**5-10 year (N=36)**	**>10 year (N=34)**	**P-value**

**FSFI total score**					
Mean (SD)	19.3 (9.8)	16.4 (10.8)	15.3 (12.3)	14.5 (11.0)	0.69
**Desire**					
Mean (SD)	3.7 (1.4)	3.0 (1.7)	3.6 (1.8)	2.5 (1.2)	0.05
**Arousal**					
Mean (SD)	2.5 (1.9)	2.0 (1.9)	2.8 (2.2)	2.0 (1.8)	0.45
**Lubrication**					
Mean (SD)	3.0 (2.3)	2.6 (2.5)	2.7 (2.5)	2.2 (2.1)	0.76
**Orgasm**					
Mean (SD)	2.8 (2.1)	2.6 (2.4)	2.3 (2.40)	2.1 (2.3)	0.83
**Satisfaction**					
Mean (SD)	3.60 (1.3)	3.2 (1.5)	2.9 (2.1)	2.9 (2.0)	0.7
**Pain**					
Mean (SD)	3.8 (2.7)	2.9 (2.6)	2.1 (2.4)	2.5 (2.6)	0.32
Sexual dysfunction	80.0%	77.8%	74.1%	85.7%	0.75
No Sexual dysfunction	20.0%	22.2%	25.9%	14.3%	

**WOMEN SEXUAL FUNCTION by disease control**	**Disease control (N=91)**	**No disease control (N=38)**	**P-value**
**FSFI total score**			
Mean (SD)	15.7 (11.1)	15.8 (11.7)	0.95
**Desire**			
Mean (SD)	3.3 (1.6)	2.5 (1.4)	0.011
**Arousal**			
Mean (SD)	2.4 (2.1)	2.2 (1.8)	0.69
**Lubrication**			
Mean (SD)	2.5 (2.3)	2.8 (2.4)	0.75
**Orgasm**			

Mean (SD)	2.3 (2.3)	2.8 (2.4)	0.33
**Satisfaction**			
Mean (SD)	2.9 (1.78)	3.2 (1.9)	0.48
**Pain**			
Mean (SD)	2.5 (2.46)	3.0 (2.6)	0.3
Sexual dysfunction	76.7 %	78.9 %	0.98
No Sexual dysfunction	23.3%	21.1 %	

**WOMEN SEXUAL FUNCTION by IGF-I class**	**<=1 (N=91)**	**1.0-1.3 (N=18)**	**>1.3 (N=20)**	**P-value**
**FSFI total score**				
Mean (SD)	15.7 (11.1)	14.2 (12.6)	17.3 (10.9)	0.7
**Desire**				
Mean (SD)	3.3 (1.6)	2.4 (1.5)	2.7 (1.3)	0.044
**Arousal**				
Mean (SD)	2.4 (2.0)	1.9 (2.0)	2.5 (1.7)	0.57
**Lubrication**				
Mean (SD)	2.5 (2.3)	2.2 (2.6)	3.0 (2.3)	0.54
**Orgasm**				
Mean (SD)	2.3 (2.3)	2.4 (2.7)	3.1 (2.1)	0.39
**Satisfaction**				
Mean (SD)	2.9 (1.8)	3.0 (2.0)	3.3 (1.8)	0.68
**Pain**				
Mean (SD)	2.5 (2.6)	2.6 (2.6)	3.4 (2.5)	0.30
Sexual dysfunction	76.7 %	77.8%	80.0 %	0.95
No Sexual dysfunction	23.3 %	22.2%	20.0 %	

Supplemental Table 6: Results of Corsi Block Tapping task , Digit Span, Trial Making Test, Phonemic Verb Fluency questionnaires

**Corsi Block Tapping Task (fw)**		Score
Mean (SD)	4.8 (1.3)	Normal score >1
Visuospatial working memory troubles	9.5 %	
No Visuospatial working memory troubles	90.5%	
**Corsi Block Tapping Task (bw)**		
Mean (SD)	3.8 (1.4)	Normal score >1
Visuospatial working memory troubles	9.5 %	
No Visuospatial working memory troubles	90.5%	
**DIGIT SPAN (fw)**		
Mean (SD)	6.0 (1.5)	Normal score >1
Short-term verbal working memory troubles	14.5 %	
No Short-term verbal working memory troubles	85.5 %	
**DIGIT SPAN (bw)**		
Mean (SD)	3.9 (1.3)	Normal score >1
Short-term verbal working memory troubles	12.7 %	
No Short-term verbal working memory troubles	87.3 %	
**Trial Making Test A**		
Mean (SD)	33.9 (17.3)	
Reduced attentive process	12.3%	
No Reduced attentive process	87.7%	
**Trial Making Test B**		
Mean (SD)	90.7 (69.5)	
Reduced attentive process	9.7%	
No Reduced attentive process	90.3%	
**Trial Making Test B-A**		
Mean (SD)	57.4 (63.8)	
Reduced attentive process	9.7%	
No Reduced attentive process	90.3%	
**PHONEMIC VERB FLUENCY**		
Mean (SD)	30.7 (9.3)	Normal score >17

Verbal fluency troubles	11.5%
No Verbal fluency troubles	88.5%

Cognitive functions by age range	<45 years (N=42)	45-64 years (N=125)	>64 years (N=56)	P-value
**Corsi Block Tapping Task (fw)**				
Mean (SD)	5.1 (1.6)	4.7 (1.3)	4.7 (1.1)	0.27
Visuospatial working memory troubles	7.3%	11.3%	7.1%	0.59
No Visuospatial working memory troubles	92.7%	88.7%	92.9%	
**Corsi Block Tapping Task (bw)**				
Mean (SD)	4.0 (1.3)	3.9 (1.5)	3.6 (1.1)	0.23
Visuospatial working memory troubles	7.3%	11.3%	7.1%	0.59
No Visuospatial working memory troubles	92.7%	88.7%	92.9%	
**DIGIT SPAN (fw)**				
Mean (SD)	6.2 (1.4)	6.0 (1.6)	5.7 (1.1)	0.29
Short-term verbal working memory troubles	12.2%	16.1%	12.5%	0.73
No Short-term verbal working memory troubles	87.8%	83.9%	87.5%	
**DIGIT SPAN (bw)**				
Mean (SD)	4.0 (1.1)	4.0 (1.65)	3.9 (1.1)	0.93
Short-term verbal working memory troubles	7.3%	15.3%	10.7%	0.36
No Short-term verbal working memory troubles	92.7 %	84.7%	89.3%	
**Trial Making Test A**				
Mean (SD)	34.7 (11.3)	32.4 (14.3)	36.7 (25.3)	0.3
Reduced attentive process	9.8%	9.7%	20.0%	0.13
No Reduced attentive process	90.2 %	90.3%	80.0%	
**Trial Making Test B**				
Mean (SD)	100.0 (58.7)	82.7 (64.5)	102.0 (86.0)	0.15
Reduced attentive process	7.3%	7.3%	17.3%	0.1
No Reduced attentive process	92.7 %	92.7 %	82.7%	

Cognitive functions by age range	<45 years (N=42)	45-64 years (N=125)	>64 years (N=56)	P-value
**Trial Making Test B-A**				
Mean (SD)	65.2 (56.4)	51.0 (58.8)	66.2 (78.4)	0.24
Reduced attentive process	9.8%	8.1%	13.5%	0.54
No Reduced attentive process	90.2 %	91.9%	86.5%	
**PHONEMIC VERB FLUENCY**				
Mean (SD)	29.0 (7.7)	32.0 (9.6)	29.1 (9.5)	0.065
Verbal fluency troubles	17.1%	9.0%	12.7%	0.35
No Verbal fluency troubles	82.9 %	91.0%	87.3%	

Cognitive functions by gender	Men (N=94)	Women (N=129)	P-value
**Corsi Block Tapping Task (fw)**			
Mean (SD)	4.8 (1.2)	4.8 (1.4)	0.9
Visuospatial working memory troubles	6.4%	11.7%	0.28
No Visuospatial working memory troubles	93.6%	88.3%	
**Corsi Block Tapping Task (bw)**			
Mean (SD)	4.1 (1.4)	3.6 (1.3)	0.028
Visuospatial working memory troubles	6.4%	11.7%	0.28
No Visuospatial working memory troubles	93.6%	88.3%	
**DIGIT SPAN (fw)**			
Mean (SD)	6.2 (1.7)	5.8 (1.3)	0.14
Short-term verbal working memory troubles	19.4%	10.9%	0.12
No Short-term verbal working memory troubles	80.6%	89.1%	
**DIGIT SPAN (bw)**			
Mean (SD)	4.2 (1.4)	3.8 (1.2)	0.051
Short-term verbal working memory troubles	10.8%	14.1%	0.6
No Short-term verbal working memory troubles	89.2%	85.9%	

Cognitive functions by gender	Men (N=94)	Women (N=129)	P-value
**Trial Making Test A**			
Mean (SD)	31.9 (17.6)	35.4 (17.0)	0.14
Reduced attentive process	7.5%	15.7%	0.1
No Reduced attentive process	92.5%	84.3%	
**Trial Making Test B**			
Mean (SD)	80.3 (60.8)	98.5 (74.7)	0.05
Reduced attentive process	5.4%	12.9%	0.1
No Reduced attentive process	94.6%	87.1%	
**Trial Making Test B-A**			
Mean (SD)	49.1 (54.0)	63.5 (69.8)	0.089
Reduced attentive process	6.4%	12.1%	0.25
No Reduced attentive process	93.6%	87.9%	
**PHONEMIC VERB FLUENCY**			
Mean (SD)	29.6 (8.9)	31.5 (9.6)	0.14
Verbal fluency troubles	12.1%	11.0%	0.98
No Verbal fluency troubles	87.9%	89.0%	

Cognitive functions by disease duration	<2 year (N=25)	2-5 year (N=55)	5-10 year (N=59)	>10 year (N=52)	P-value
**Corsi Block Tapping Task (fw)**					
Mean (SD)	5.0 (0.9)	5.0 (1.4)	4.5 (1.3)	4.8 (1.3)	0.19
Visuospatial working memory troubles	0.0%	9.1%	14.0 %	7.7 %	0.23
No Visuospatial working memory troubles	100.0%	90.9%	86.0 %	92.3%	
**Corsi Block Tapping Task (bw)**					
Mean (SD)	4.3 (1.6)	4.0 (1.42)	3.7 (1.5)	3.7 (1.3)	0.17
Visuospatial working memory troubles	0.0%	9.1%	14.0 %	7.7 %	0.23
No Visuospatial working memory troubles	100.0%	90.9%	86.0 %	92.3%	
**DIGIT SPAN (fw)**					

Cognitive functions by disease duration	<2 year (N=25)	2-5 year (N=55)	5-10 year (N=59)	>10 year (N=52)	P-value
Mean (SD)	6.2 (1.4)	6.0 (1.3)	6.0 (1.4)	5.9 (1.6)	0.382
Short-term verbal working memory troubles	12.0%	12.7 %	12.3 %	17.3 %	0.86
No Short-term verbal working memory troubles	88.0%	87.3 %	87.7 %	82.7 %	
**DIGIT SPAN (bw)**					
Mean (SD)	4.3 (1.5)	4.0 (1.0)	4.0 (1.2)	4.0 (1.5)	0.6
Short-term verbal working memory troubles	8.0%	9.1 %	8.8 %	13.5 %	0.81
No Short-term verbal working memory troubles	92.0%	90.9 %	91.2 %	86.5 %	
**Trial Making Test A**					
Mean (SD)	33.4 (11.0)	36.1 (15.0)	34.2 (23.6)	32.2 (14.7)	0.72
Reduced attentive process	4.0%	14.8 %	15.8 %	11.5 %	0.48
No Reduced attentive process	96.0%	85.2 %	84.2 %	88.5 %	
**Trial Making Test B**					
Mean (SD)	81.5 (28.9)	88.4 (49.6)	90.8 (77.5)	91.2 (79.0)	0.94
Reduced attentive process	0.0%	5.6 %	16.1%	13.7 %	0.34
No Reduced attentive process	100.0%	94.4 %	83.9 %	86.3 %	
**Trial Making Test B-A**					
Mean (SD)	47.6 (24.7)	52.8 (46.4)	57.6 (68.5)	60.4 (76.5)	0.83
Reduced attentive process	4.2%	5.6 %	12.5%	13.7 %	0.34
No Reduced attentive process	95.8 %	94.4 %	87.5 %	86.3 %	
**PHONEMIC VERB FLUENCY**					
Mean (SD)	32.0 (6.9)	30.5 (8.3)	32 (10.6)	30.3 (9.7)	0.74

Cognitive functions by disease duration	<2 year (N=25)	2-5 year (N=55)	5-10 year (N=59)	>10 year (N=52)	P-value
Verbal fluency troubles	4.0 %	7.3 %	10.7%	16.0%	0.33
No Verbal fluency troubles	96.0 %	92.7 %	89.3%	84.0%	

Cognitive functions disease control	Disease control (N=156)	No disease control (N=67)	P-value
**Corsi Block Tapping Task (fw)**			
Mean (SD)	4.8 (1.3)	4.8 (1.2)	0.85
Visuospatial working memory troubles	10.4%	7.5%	0.76
No Visuospatial working memory troubles	89.6%	92.5%	0.67
**Corsi Block Tapping Task (bw)**			
Mean (SD)	3.7 (1.3)	4.1 (1.6)	0.095
Visuospatial working memory troubles	10.4%	7.5%	0.67
No Visuospatial working memory troubles	89.6%	92.5%	
**DIGIT SPAN (fw)**			
Mean (SD)	5.9 (1.5)	6.1 (1.4)	0.34
Short-term verbal working memory troubles	15.6%	11.9%	0.62
No Short-term verbal working memory troubles	84.4%	88.1%	
**DIGIT SPAN (bw)**			
Mean (SD)	3.9 (1.3)	4.0 (1.4)	0.76
Short-term verbal working memory troubles	12.3%	13.4%	0.99
No Short-term verbal working memory troubles	87.7%	86.6%	
**Trial Making Test A**			
Mean (SD)	34.1 (17.3)	33.5 (17.5)	0.81
Reduced attentive process	11.8%	13.4%	0.9
No Reduced attentive process	88.2%	86.6%	

Cognitive functions disease control	Disease control (N=156)	No disease control (N=67)	P-value
**Trial Making Test B**			
Mean (SD)	87.9 (67.8)	97.2 (73.4)	0.38
Reduced attentive process	9.3%	10.6%	0.96
No Reduced attentive process	90.7%	89.4%	
**Trial Making Test B-A**			
Mean (SD)	54.3 (63.0)	64.2 (65.3)	0.3
Reduced attentive process	8.6%	12.1%	0.58
No Reduced attentive process	91.4%	87.9%	
**PHONEMIC VERB FLUENCY**			
Mean (SD)	30.4 (9.4)	31.3 (9.2)	0.51
Verbal fluency troubles	11.7%	10.9%	0.99
No Verbal fluency troubles	88.3%	89.1%	

Cognitive functions by IGF-I categories	<=1 (N=156)	1.0-1.3 (N=32)	>1.3 (N=35)	P-value
**Corsi Block Tapping Task (fw)**				
Mean (SD)	4.8 (1.3)	4.8 (1.3)	4.8 (1.1)	0.98
Visuospatial working memory troubles	10.4%	6.2%	8.6%	0.75
No Visuospatial working memory troubles	89.6%	93.8%	91.4%	
**Corsi Block Tapping Task (bw)**				
Mean (SD)	3.7 (1.3)	3.9 (1.7)	4.3 (1.6)	0.09
Visuospatial working memory troubles	10.4%	6.2%	8.6%	0.75
No Visuospatial working memory troubles	89.6%	93.8%	91.4%	
**DIGIT SPAN (fw)**				
Mean (SD)	5.9 (1.5)	6.2 (1.6)	6.1 (1.2)	0.62
Short-term verbal working memory troubles	15.6%	12.5%	11.4%	0.77
No Short-term verbal working memory troubles	84.4%	87.5%	88.6%	
**DIGIT SPAN (bw)**				
Mean (SD)	3.9 (1.3)	3.7 (1.5)	4.3 (1.2)	0.2

**Cognitive functions by IGF-I categories**	**<=1 (N=156)**	**1.0-1.3 (N=32)**	**>1.3 (N=35)**	**P-value**
Short-term verbal working memory troubles	12.3%	21.9%	5.7%	0.14
No Short-term verbal working memory troubles	87.7%	78.1%	94.3%	
**Trial Making Test A**				
Mean (SD)	34.1 (17.3)	35.2 (18.8)	32.0 (16.4)	0.73
Reduced attentive process	11.8%	15.6%	11.4%	0.82
No Reduced attentive process	88.2%	84.4%	88.6%	
**Trial Making Test B**				
Mean (SD)	87.9 (67.8)	85.2 (42.5)	108 (91.8)	0.26
Reduced attentive process	9.3%	6.2%	14.7%	0.49
No Reduced attentive process	90.7%	93.8%	85.3%	
**Trial Making Test B-A**				
Mean (SD)	54.3 (63.0)	49.5 (39.3)	78.1 (81.0)	0.11
Reduced attentive process	8.6%	6.2%	17.6%	0.21
No Reduced attentive process	91.4%	93.8%	82.4%	
**PHONEMIC VERB FLUENCY**				
Mean (SD)	30.4 (9.4)	30.7 (7.6)	32.0 (10.6)	0.69
Verbal fluency troubles	11.7%	6.2%	15.6%	0.49
No Verbal fluency troubles	88.3%	93.8%	84.4%	

Supplemental Table 7: Results of the AcroQol

**ACROQoL**	
**Total score**	
Mean (SD)	62.4 (20.4)
**Physical Quality**	
Mean (SD)	56.5 (25.3)
**Psychological Quality**	
Mean (SD)	64.5 (19.7)
**Body Image Quality**	
Mean (SD)	54.3 (24.0)
**Social Relationship Quality**	
Mean (SD)	75.1 (19.3)

**ACROQoL by age class**	**<45 years (N=42)**	**45-64 years (N=125)**	**>64 years (N=56)**	**P-value**
**Total score**				
Mean (SD)	61.9 (19.3)	61.7 (21.4)	64.6 (18.9)	0.69
**Physical Quality**				
Mean (SD)	55.8 (22.9)	57.0 (25.9)	55.9 (26.1)	0.95
**Psychological Quality**				
Mean (SD)	62.8 (18.5)	63.3 (21.0)	68.6 (17.2)	0.23
**Body Image Quality**				
Mean (SD)	53.6 (21.1)	53.6 (25.0)	56.4 (24.1)	0.77
**Social Relationship Quality**				
Mean (SD)	72.0 (19.3)	73.8 (20.6)	80.8 (15.1)	0.05

**ACROQoL by gender**	**Male (N=94)**	**Female (N=129)**	**P-value**
**Total score**			
Mean (SD)	64.1 (21.8)	61.2 (19.2)	0.31

**Physical Quality**			
Mean (SD)	58.5 (27.1)	55.0 (23.8)	0.33
**Psychological Quality**			
Mean (SD)	66.0 (20.9)	63.3 (18.8)	0.33
**Body Image Quality**			
Mean (SD)	56.5 (25.2)	52.6 (23.1)	0.25
**Social Relationship Quality**			
Mean (SD)	76.3 (19.9)	74.2 (18.9)	0.43

**ACROQoL by disease duration**	**<2 year (N=25)**	**2-5 year (N=55)**	**5-10 year (N=59)**	**10+ year (N=52)**	**P-value**
**Total score**					
Mean (SD)	60.6 (24.9)	60.1 (19.2)	64.4 (19.1)	62.4 (21.1)	0.72
**Physical Quality**					
Mean (SD)	53.6 (26.9)	54.3 (25.0)	59.6 (25.7)	56.7 (24.7)	0.67
**Psychological Quality**					
Mean (SD)	60.8 (24.0)	63.1 (17.7)	65.5 (17.8)	64.6 (21.7)	0.79
**Body Image Quality**					
Mean (SD)	52.9 (28.0)	51.1 (22.4)	55.6 (22.3)	54.0 (25.9)	0.81
**Social Relationship Quality**					
Mean (SD)	68.9 (22.8)	75.5 (16.5)	76.3 (18.6)	75.5 (21.9)	0.43

**ACROQoL by disease control**	**Disease control (N=156)**	**No disease control (N=67)**	**P-value**
**Total score**			
Mean (SD)	62.7 (20.2)	61.9 (20.9)	0.81
**Physical Quality**			
Mean (SD)	57.1 (25.0)	55.3 (26.1)	0.65
**Psychological Quality**			
Mean (SD)	65.0 (19.7)	63.2 (19.9)	0.53

**Body Image Quality**			
Mean (SD)	55.1 (23.8)	52.5 (24.6)	0.48
**Social Relationship Quality**			
Mean (SD)	75.2 (19.3)	74.9 (19.4)	0.93

ACROQoL by IGF-I class	<=1 (N=156)	1.0-1.3 (N=32)	>1.3 (N=35)	P-value
**Total score**				
Mean (SD)	62.7 (20.2)	59.6 (21.1)	66.3 (20.1)	0.18
**Physical Quality**				
Mean (SD)	57.1 (25.0)	48.6 (25.94)	61.3 (25.6)	0.12
**Psychological Quality**				
Mean (SD)	65.0 (19.7)	57.7 (20.3)	68.0 (18.6)	0.091
**Body Image Quality**				
Mean (SD)	55.1 (23.8)	44.7 (23.6)	59.3 (23.7)	0.039
**Social Relationship Quality**				
Mean (SD)	75.2 (19.3)	70.7 (20.9)	78.6 (17.6)	0.27
